# LBX2-AS1 as a Novel Diagnostic Biomarker and Therapeutic Target Facilitates Multiple Myeloma Progression by Enhancing mRNA Stability of LBX2

**DOI:** 10.3389/fmolb.2021.706570

**Published:** 2021-09-06

**Authors:** Haipeng Jia, Yan Liu, Sulong Lv, Ruifang Qiao, Xiaofen Zhang, Fei Niu, Wenqing Shang, Shumei Liu, Jing Dong, Zhirong Zhang

**Affiliations:** ^1^ Department of Hematology, The Second Affiliated Hospital of Shandong First Medical University, Tai’an, China; ^2^ Respiratory Intensive Care Unit, Tai’an City Central Hospital, Tai’an, China; ^3^ Department of Emergency, The Second Affiliated Hospital of Shandong First Medical University, Tai’an, China

**Keywords:** multiple myeloma, LBX2-AS1, LBX2, mRNA stability, proliferation, apoptosis

## Abstract

**Objective:** Multiple myeloma (MM) represents a common age-associated malignancy globally. The function and underlying mechanism of antisense lncRNA LBX2-AS1 remain ambiguous in multiple myeloma (MM). Herein, we aimed to observe the biological implication of this lncRNA in MM.

**Methods:** RT-qPCR was employed to examine circulating LBX2-AS1 and LBX2 in 60 paired MM and healthy subjects. Correlation between the two was analyzed by Pearson test. Under transfection with shLBX2-AS1, proliferation and apoptosis were evaluated in MM cells through CCK-8, colony formation and flow cytometry. LBX2 expression was examined in MM cells with shLBX2-AS1 or pcDNA3.1-LBX2 transfection. Following treatment with cycloheximide or actinomycin D, LBX2 expression was examined in pcDNA3.1-LBX2-transfected MM cells at different time points. Rescue assays were then presented. Finally, xenograft tumor models were established.

**Results:** Circulating LBX2-AS1 was up-regulated in MM patients and positively correlated to LBX2 expression. Area under the curve (AUC) of LBX2-AS1 expression was 0.7525. Its up-regulation was also found in MM cells and primarily distributed in cytoplasm. LBX2-AS1 knockdown distinctly weakened proliferative ability and induced apoptosis in MM cells. Overexpressing LBX2-AS1 markedly strengthened LBX2 expression by increasing its mRNA stability. Rescue assays showed that silencing LBX2-AS1 distinctly weakened the pcDNA3.1-LBX2-induced increase in proliferation and decrease in apoptosis for MM cells. Silencing LBX2-AS1 markedly weakened tumor growth.

**Conclusion:** Our data demonstrated that circulating LBX2-AS1 could be an underlying diagnostic marker in MM. Targeting LBX2-AS1 suppressed tumor progression by affecting mRNA stability of LBX2 in MM. Hence, LBX2-AS1 could be a novel therapeutic marker against MM.

## Introduction

Multiple myeloma (MM), a B-cell malignant disease, has a feature with aberrant proliferative capacities of plasmocytes in the bone marrow (Wang et al., 2020). Stem cell transplantation, radiotherapy, chemotherapy as well as targeted therapy are the main treatment strategies for MM (Pan et al., 2019). However, the survival outcome remains undesirable. The 5-years overall survival rate is approximately 45% (Amodio et al., 2018). Hence, in-depth research is warranted to probe into the molecular mechanisms underlying MM to develop more favorable therapeutic strategies against MM.

The occurrence and progression of MM is the result of the joint action of genetic and environmental factors (Foltz et al., 2020). At the cellular level, it shows the uncontrollability of cell growth and proliferation, while it shows overexpressed oncogenes as well as inactivated tumor suppressor genes at the genetic level (Szcześniak et al., 2020). Long non-coding RNA (LncRNA) is a type of RNA with a length of >200 nucleotides, most of which do not have the capacity to encode proteins (Arun et al., 2018). LncRNA is specifically distributed in different tissues or cancer cells (Statello et al., 2021). LBX2 antisense RNA 1 (LBX2-AS1), a novel lncRNA, exerts an oncogenic role in different malignancies. For instance, LBX2-AS1 is up-regulated in gastric cancer and silencing LBX2-AS1 restrains proliferative, migratory, and invasive capacities of gastric cancer cells by miR-491-5p/ZNF703, miR-219a-2-3p/FUS or miR-4766-5p/CXCL5 axis (Peng et al., 2020; Xu et al., 2020; Yang et al., 2020). High expression of LBX2-AS1 is also reported in ovarian cancer and its up-regulation is in relation to undesirable survival outcome (Cao et al., 2021). LBX2-AS1 may promote ovarian cancer progression through miR-455-5p/E2F2, miR-491-5p/E2F2 (Cao et al., 2021) and miR-4784/KDM5C axis (Gu et al., 2021). Furthermore, LBX2-AS1 that can be activated through ZEB1, accelerates migration and epithelial-mesenchymal transition in esophageal squamous cell carcinoma *via* interaction with HNRNPC, thereby stabilizing ZEB1 as well as ZEB2 (Zhang et al., 2019). LBX2-AS1 up-regulation exhibits correlation to the staging, metastasis, and prognosis of hepatocellular carcinoma patients (Wang et al., 2020c). It may drive hepatocellular carcinoma progression *via* miR-384/IRS1 pathway (Wang et al., 2020c). Also, this lncRNA accelerates proliferation as well as metastases *via* Notch pathway in non-small cell lung cancer (Tang et al., 2019). Given the previous findings, LBX2-AS1 plays critical roles in carcinogenesis. Nevertheless, its function and mechanism in the progression of MM remain undetermined. Herein, we observed the clinical implications and biological roles of LBX2-AS1 in MM, which might contribute to therapeutic improvement in MM.

## Materials and Methods

### Patients

From January 2018 to December 2019, 60 patients with MM who were newly diagnosed in The Second Affiliated Hospital of Shandong First Medical University were included in this study. Inclusion criteria were as follows: 1) The diagnosis of MM complied with the 2003 International Myeloma Working Group (2003) (IMWG) MM diagnostic criteria (2003); 2) patients had complete clinical information; 3) patients were newly diagnosed as MM and did not receive any treatment. Exclusion criteria were as follows: 1) Patients combined with malignant tumor history or treatment history; 2) patients combined with leukemia, lymphoma, and other blood system diseases; 3) patients had infectious diseases such as pneumonia and tuberculosis; 4) patients had neuropsychiatric disorders who cannot cooperate with treatment; 5) patients received other treatments in the past. There were 32 males and 28 females. Average age was 56.3 ± 7.2. According to the International Staging System (ISS) (Greipp et al., 2005), there were 14 cases with stage I (β2-MG ≤ 3.5 mg/L and albumin >35 g/L), 20 cases with stage II (3.5 mg/L < β2-MG < 5.5 mg/L), 26 cases with stage III (β2-MG ≥ 5.5 mg/L). According to DS staging, there were 13 cases of stage I, 13 cases of stage II, and 34 cases of stage III. Diagnosis types were as follows: 34 cases of IgG type, 15 cases of IgA type, 11 cases of light chain type and other types. Hemoglobin of 38 cases was <100 g/L and 22 cases ≥100 g/L. Blood calcium of 39 cases was <2.98 mmol/L and 21 cases ≥2.98 mmol/L. There were 36 cases with lactate dehydrogenase <245 U/L and 24 cases with ≥245 U/L. 60 healthy people who received physical examination during the same period served as controls, including 30 males and 30 females. Average age was 57.6 ± 5.5. This study was approved by the Ethics Committee of The Second Affiliated Hospital of Shandong First Medical University (2021-007). All patients signed the written informed consent.

### Blood Specimen Collection

5 ml of venous blood was collected on fasting in the morning on the day of admission. Blood samples were placed at room temperature for 30 min. Then, samples were centrifuged at 3,000 r/min for 10 min. After the centrifugation, samples were transferred to a new centrifuge tube and stored in −80°C ultra-low temperature refrigerator.

### Real-Time Quantitative PCR

Total RNA was extracted from serum specimens, cells, or tissues by Trizol reagent. 1 μg RNA was used as a template for reverse transcription to synthesize cDNA. The SYBR Premix Ex Taq Kit (DRR041; TaKaRa) was used for RT-qPCR on the ABI PRISM 7900 qRT-PCR instrument (ABI, United States). The reaction system contained 2 μl 5 × primerScript Buffer, 1 μl PrimerScript RT Enzyme Mix Ⅰ, 10 μl template, 1 μl neck loop primer and DEPC water. Total reaction system was as follows: 10 μl 2 × Master Mix, 1 μl forward and reverse primers, 1 μl cDNA, and 7 μl RNase-free water. The reaction conditions were as follows: at 95°C for 2 min, at 95°C for 20 s, at 60°C for 20 s, at 70°C for 20 s, a total of 39 cycles. The primer sequences were as follows: LBX2-AS1: 5′-CGT​GGG​GAA​TGG​ACC​CAT​AG-3′ (forward), 5′-GGA​CTT​GCC​CTT​GGT​GAC​TC-3′ (reverse); LBX2: 5′-TCC​AGG​GCG​GAA​AAC​AAC​TC-3′ (forward), 5′-GTG​CTA​AGC​TGC​ACA​GGA​CT-3′ (reverse); β-actin: 5′-CTC​CAT​CCT​GGC​CTC​GCT​GT-3′ (forward), 5′-GCT​GTC​ACC​TTC​ACC​GTT​CC-3′ (reverse); 18S rRNA: 5′-GTA​ACC​CGT​TGA​ACC​CCA​TT-3′ (forward), 5′-CCA​TCC​AAT​CGG​TAG​TAG​CG-3′ (reverse); U6: 5ʹ-CTC​GCT​TCG​GCA​GCA​CA-3ʹ (forward), 5ʹ-AAC​GCT​TCA​CGA​ATT​TGC​GT-3ʹ (reverse). Relative expression was determined with the 2^−ΔΔCt^ method.

### Cell Culture

Human MM cell lines NCI-H929 and U266 were retrieved from the American Type Culture Collection. They were cultured in RPMI medium 1,640 (118,75127; Gibco, Carlsbad, CA, United States) plus 10% fetal bovine serum (164,210-500; Gibco) and 1% penicillin–streptomycin at 37°C in a humidified incubator of 5%. Furthermore, normal human marrow CD138^+ ^plasmocytes were acquired through CD138 magnetic beads (18,000; Stem cell, United States).

### Subcellular Fractionation Assay

Using the cytoplasmic and nuclear RNA purification kit (Ontario, Canada), LBX2-AS1 expression in cytoplasm as well as nucleus was detected through RT-qPCR. U6 and 18S separately served as the nuclear and cytoplasmic controls.

### Transfection

The shRNA negative control (shNC): 5′-CCG​CCT​TAA​TGT​GCA​ATA​AAG​CAG​CCT​CGA​GGC​TGC​TTT​ATT​GCA​CAT​TAA​GTT​TTT​G-3′, shLBX2-AS1#1: 5′-CCG​CCA​AGT​TAT​AAA​ACT​ATA​ATG​CCT​CGA​GGC​ATT​ATA​GTT​TTA​TAA​CTT​GTT​TTT​G-3′, shLBX2-AS1#2: 5′-CCG​CGG​AAT​GTT​TGC​TGA​ATT​AAT​GCT​CGA​GCA​TTA​ATT​CAG​CAA​ACA​TTC​CTT​TTT​G-3′, pcDNA3.1 vectors expressing LBX2-AS1 and LBX2 as well as empty vectors were purchased from Genechem (Shanghai, China). The well-growing MM cells were seeded on a 6-well plate (2 × 10^5^/well). When cells reached 80% confluence, 100 pmol transfection plasmid and 5 μl transfection reagent lipofectamine 2,000 were diluted with serum-free Opti-MEM medium. After mixing the two, they were incubated at room temperature lasting 5 min. Then, cells were separately transfected into sh-NC, shLBX2-AS1#1 and shLBX2-AS1#2. After 48 h, the cells were collected for next assays.

### Cell Counting Kit-8

The density of MM cells was adjusted to 2.5 × 10^4^ cells/ml. 100 μl cell suspension per well was seeded in a 96-well plate. Then, cells were incubated in an incubator at 37°C and 5% CO_2_. After the cells adhered to the wall, the transfected cells were cultured for 48 h. 10 μl CCK-8 solution (CK04; DOJINDO, Japan) was then added to each well. The cells were incubated for 2 h. The absorbance value at 450 nm wavelength was measured at 0, 24, 48, and 72 h utilizing the automatic microplate reader.

### Clone Formation Assay

The transfected NCI-H929 as well as U266 cells were seeded in 6-well plates (1 × 10^5^ cells/well). After culturing for 14 days, the old medium was removed. Samples were washed using pre-cooled PBS. The cells were incubated by methanol (500 μl/well) at −20°C for 20 min. Subsequently, the cells were stained by 1% crystal violet staining solution lasting 15 min (400 μl/well). The number of clones was counted.

### Flow Cytometry

The transfected cells were centrifuged and resuspended by binding buffer. Then, they were subsequently stained using 10 μl fluorescein isothiocyanate (Annexin V-FITC) solution and 10 μl propidium iodide (PI) solution. The stained cells were incubated in the dark lasting 30 min. Then, the apoptotic levels were detected by the FACSCalibur^TM^ flow cytometer (1,026; BD, United States).

### Western Blot

The transfected NCI-H929 and U266 cells were fully lysed by RIPA lysis buffer on the ice for 30 min. The sample was centrifuged at 12,000 g for 15 min at 4°C. The supernatant was obtained and the protein content was determined by BCA method. The amount of protein loaded in each well was 40 μg. The loading buffer was added and boiled for 5 min to denature the protein. The denatured protein was taken and used for SDS-polyacrylamide gel electrophoresis. The electrophoresis voltage was 90 V constant voltage electrophoresis for 0.5 h, and then 120 V constant voltage electrophoresis for 2 h. The NC film was cut to the same size as the separation glue, and the film was transferred under a constant voltage of 60 V. The transfer device was performed at 4°C, and the transfer time was set to 60 min. The sample was sealed with 5% skimmed milk at room temperature for 2 h. Then, the PVDF membrane (Millipore, United States) was incubated with the diluted primary antibody against Bax (1/3,000; ab263897), Bcl-2 (1/2,000; ab196495), cleaved caspase3 (1/500; ab2302), LBX2 (ab164764) and GAPDH (1/10,000; ab181602) overnight at 4°C. After washing with PBS solution, horseradish peroxidase-labeled secondary antibody IgG (2985S; Cell Signaling Technology, United States) was added to the membrane, and incubated for 1 h at room temperature. Then, the membrane was added by ECL developer solution to protect from light for development. The gel imaging system was used to take protein bands. Image Pro Plus 6.0 software was employed to analyze the absorbance value of the protein bands. Target proteins were quantified with GAPDH as an internal control.

### Cycloheximide Assay

The transfected NCI-H929 and U266 cells were incubated by 100 μg/ml CHX (Sigma-Aldrich, United States). The expression of LBX2 protein was detected at 0, 2, 4, 6, and 8 h using western blot. GAPDH was used as a control.

### Actinomycin D Assay

The transfected NCI-H929 and U266 cells were treated with Actinomycin D (Sigma-Aldrich). The expression of LBX2 was detected at 0, 8, 16, and 24 h by RT-qPCR. β-actin served as a control.

### Xenografts Experiments

Totally, 24 male BALB/c nude mice with 5-week-old were purchased from Shanghai Slack Laboratory Animal Co., Ltd. (China). All mice were raised under independent ventilation cages. Xenograft tumor models were constructed in nude mice through subcutaneously injecting NCI-H929 cells that were transfected with shNC, shLBX2-AS1, empty vector and LBX2-AS1 overexpression in the armpit (6 mice in each group). After 5 weeks, tumor tissues were removed from euthanized mice. Moreover, tumor volume was measured each week. All animal experiments were carried out following the Guide for the Care and Use of Laboratory Animals of the National Institutes of Health. This project gained the approval by the Animal Care Committee of The Second Affiliated Hospital of Shandong First Medical University (2021-007).

### Statistical Analysis

Graphpad Prism 8.0.1 was employed for statistical analysis. Data were presented as the mean ± standard deviation. Comparisons between two groups were assessed through unpaired student’s t test. Meanwhile, multiple comparisons were presented by ANOVA followed by Tukey’s test. Receiver operating characteristic curve (ROC) was conducted for evaluating the diagnostic efficacy of circulating LBX2-AS1 on MM. Area under the curve (AUC) was then calculated. Pearson correlation was presented between LBX2-AS1 and LBX2 expression in 60 MM serum samples. *p* < 0.05 indicated that the difference was statistically significant.

## Results

### Circulating LBX2-AS1 is Up-Regulated in MM and Becomes a Diagnostic Marker of MM

**FIGURE 1:**
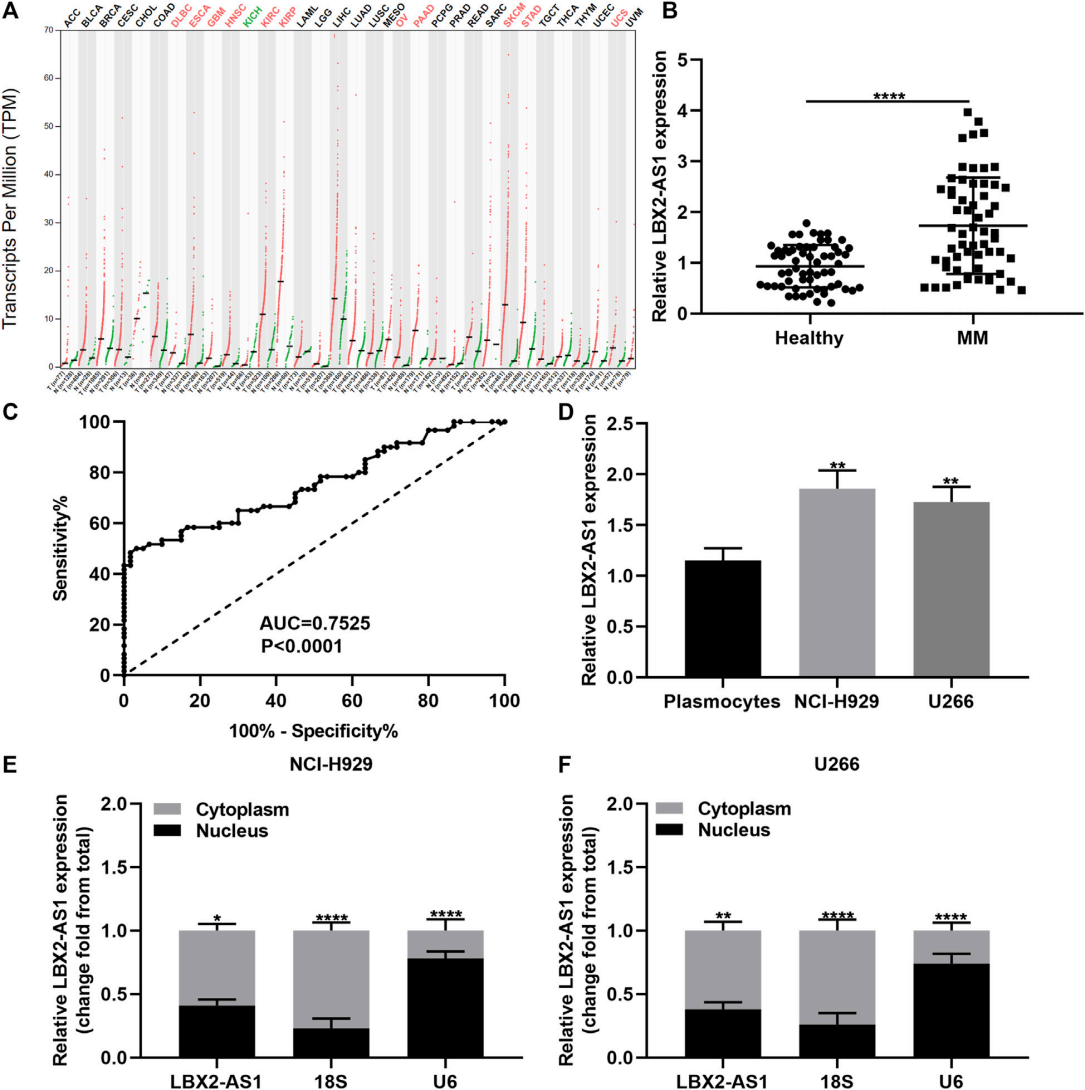
LBX2-AS1 is highly expressed in MM and could be a diagnostic marker of MM. **(A)** LBX2-AS1 expression in various cancers by the GEPIA online database. **(B)** RT-qPCR for LBX2-AS1 expression in serum samples from 60 paired MM and healthy subjects. **(C)** ROC of circulating LBX2-AS1 expression. **(D)** RT-qPCR for LBX2-AS1 expression in plasmocytes, NCI-H929 and U266 cells. **(E, F)** RT-qPCR for LBX2-AS1 expression in cytoplasm and nucleus of NCI-H929 and U266 cells. *p < 0.05; **p < 0.01; ****p < 0.0001.

From the GEPIA online database (http://gepia2.cancer-pku.cn/), LBX2-AS1 is up-regulated in various cancers (Figure 1A). Herein, we detected LBX2-AS1 expression in serum samples from 60 paired MM and healthy subjects. In Figure 1B, circulating LBX2-AS1 exhibited significantly elevated expression in MM than healthy samples (*p* < 0.0001). Furthermore, we assessed the diagnostic potential of circulating LBX2-AS1 expression in MM by ROC. The AUC value was 0.7525, indicating that LBX2-AS1 could become an effective diagnostic marker of MM (Figure 1C). LBX2-AS1 expression was also examined in MM cells as well as CD138^+^ plasmocytes. As a result, higher LBX2-AS1 expression was detected in NCI-H929 and U266 cells in comparison to plasmocytes (Figure 1D; both *p* < 0.01). LBX2-AS1 was mainly distributed in the cytoplasm and a small amount in the nucleus both in NCI-H929 (*p* < 0.05; Figure 1E) and U266 cells (*p* < 0.01; Figure 1F), suggesting that LBX2-AS1 could exert a post-transcriptional regulatory function.

### Silencing LBX2-AS1 Restrains Proliferative Capacity of MM Cells

**FIGURE 2:**
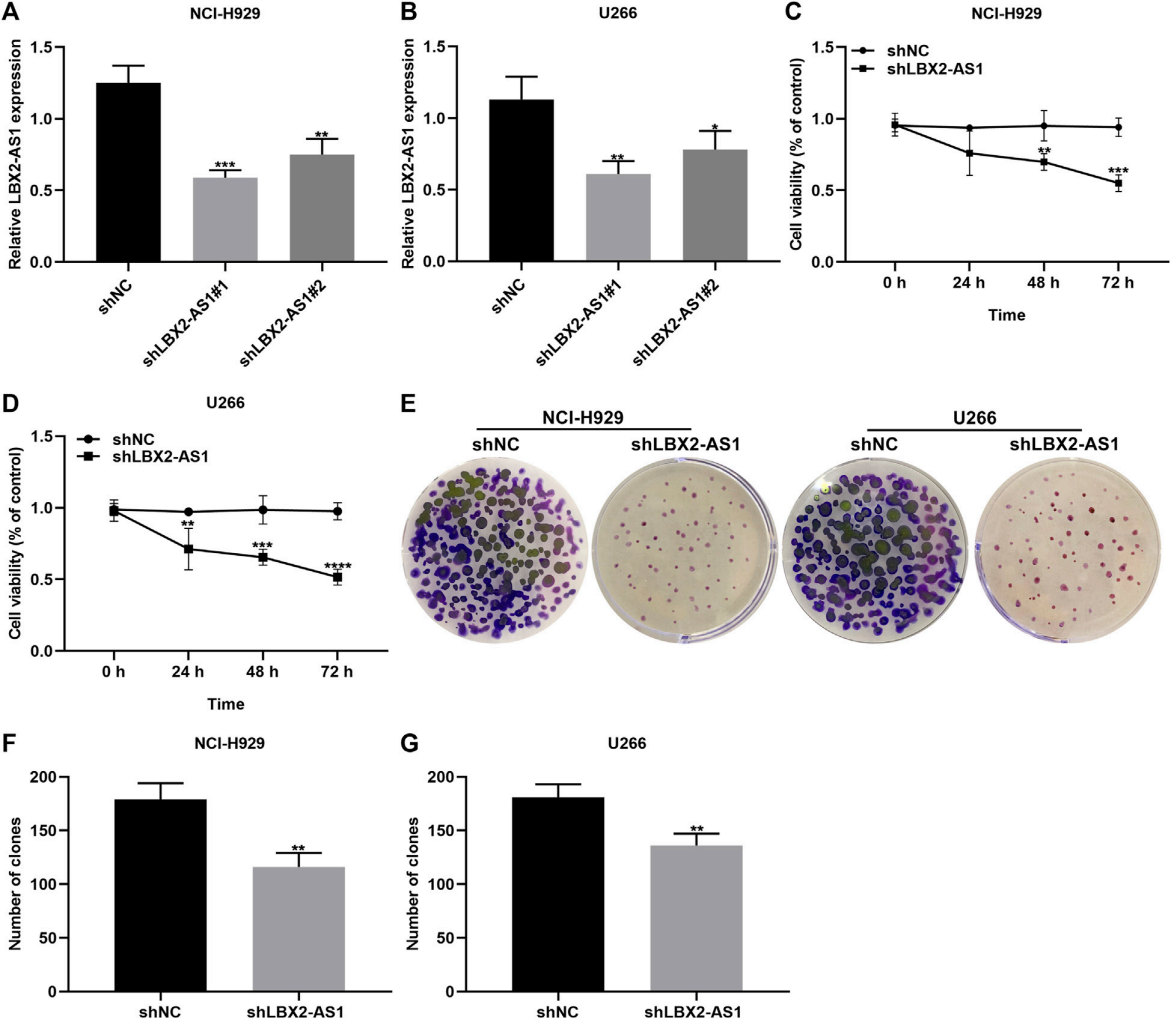
Knockdown of LBX2-AS1 suppresses proliferative ability of MM cells. **(A, B)** Detection of LBX2-AS1 expression in NCI-H929 and U266 cells transfected with shNC, shLBX2-AS1#1 or shLBX2-AS1#2 by RT-qPCR. **(C, D)** Assessment of the cell viability of MM cells with shNC or shLBX2-AS1 at 0, 24, 48, and 72 h by CCK-8 assay. **(E–G)** The number of clones for MM cells transfected with shNC or shLBX2-AS1. *p < 0.05; **p < 0.01; ***p < 0.001; ****p < 0.0001.

Two shRNAs targeting LBX2-AS1 were transected into NCI-H929 and U266 cells. RT-qPCR was applied for evaluation of the transfection effects. Our data showed that LBX2-AS1 expression was distinctly lowered by shLBX2-AS1 in NCI-H929 (*p* < 0.001 and *p* < 0.01; Figure 2A) and U266 cells (*p* < 0.01 and *p* < 0.05; Figure 2B). Then, we evaluated the proliferative capacity of MM cells following transfection with shLBX2-AS1 by applying CCK-8 and clone formation assays. Compared to shNC, cell viability was markedly decreased in NCI-H929 (Figure 2C) as well as U266 (Figure 2D) cells with shLBX2-AS1 transfection as time went by. Clone formation ability was also investigated. As a result, both in NCI-H929 (Figures 2E,F) and U266 (Figure 2G) cells, shLBX2-AS1 transfection distinctly reduced the number of clones (both *p* < 0.01). Hence, silencing LBX2-AS1 restrained proliferative capacity of MM cells.

### Silencing LBX2-AS1 Promotes Apoptosis of MM Cells

**FIGURE 3:**
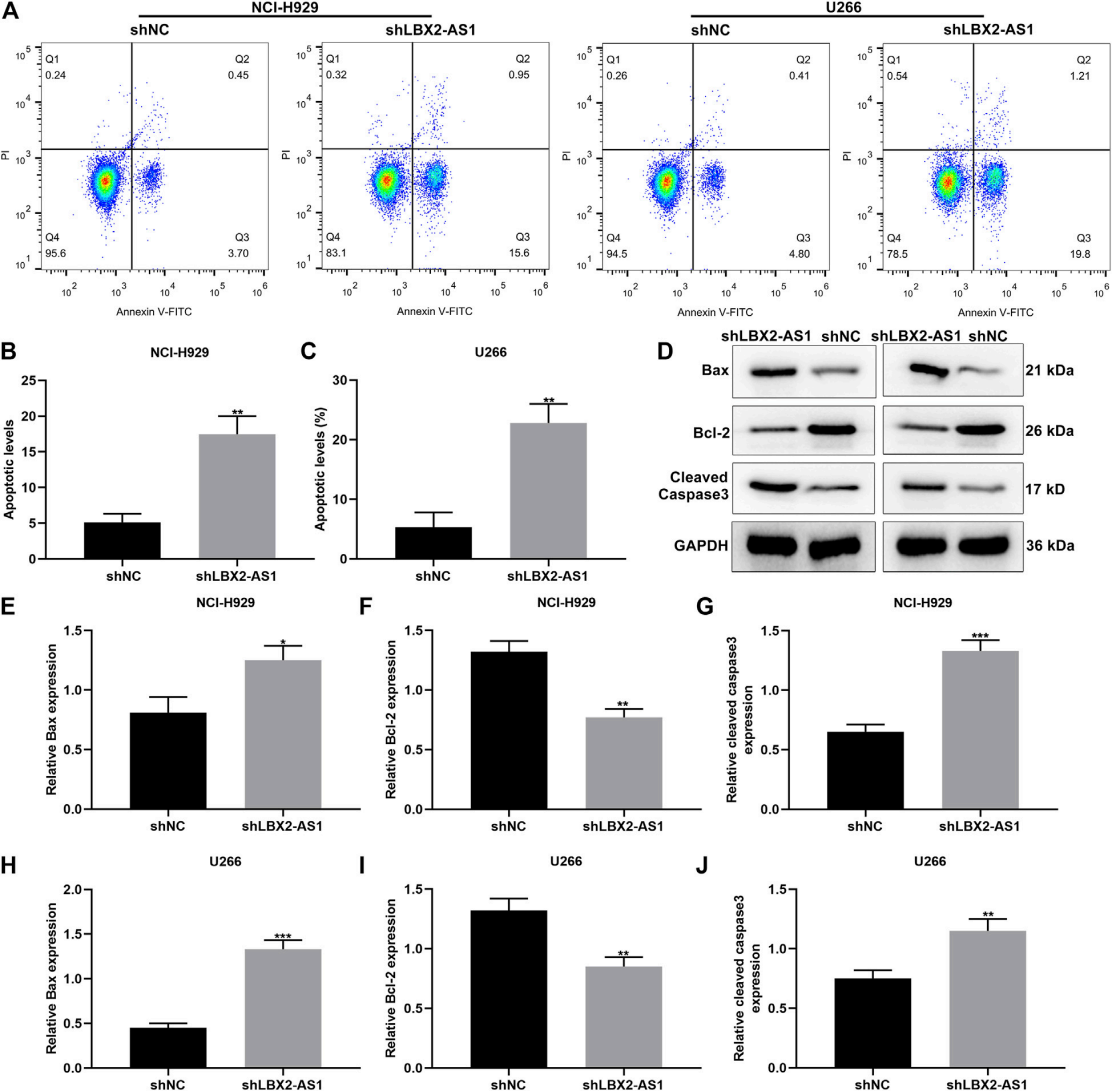
LBX2-AS1 knockdown accelerates apoptosis of MM cells. **(A–C)** The apoptotic rate of NCI-H929 and U266 cells under transfection by shNC and shLBX2-AS1 using flow cytometry assay. **(D)** Representative images of protein bands. **(E–J)** The expression of Bax, Bcl-2 and cleaved Caspase3 proteins in **(E–G)** NCI-H929 and **(H–J)** U266 cells under transfection by shNC and shLBX2-AS1 *via* western blot. *p < 0.05; **p < 0.01; ***p < 0.001.

We further evaluated the function of shLBX2-AS1 on apoptotic levels of MM cells *via* flow cytometry assay. Consequently, shLBX2-AS1 transfection distinctly induced the apoptotic rates of NCI-H929 (Figures 3A,B) and U266 cells (Figure 3C; both *p* < 0.01). Also, we detected the expression of apoptosis-related markers including Bax, Bcl-2 and cleaved Caspase3 in MM cells transfected with shLBX2-AS1 or shNC utilizing western blot (Figure 3D). Our data displayed the increase in Bax expression (*p* < 0.05; Figure 3E), the reduction in Bcl-2 expression (*p* < 0.01; Figure 3F) and the increase in cleaved Caspase3 expression (*p* < 0.001; Figure 3G) in NCI-H929 cells transfected with shLBX2-AS1 in comparison to shNC. The similar results were investigated in U266 cells (Figures 3H–J). Collectively, silencing LBX2-AS1 may induce apoptosis of MM cells.

### Serum LBX2 is Up-Regulated in MM and Positively Correlated to LBX2-AS1

**FIGURE 4:**
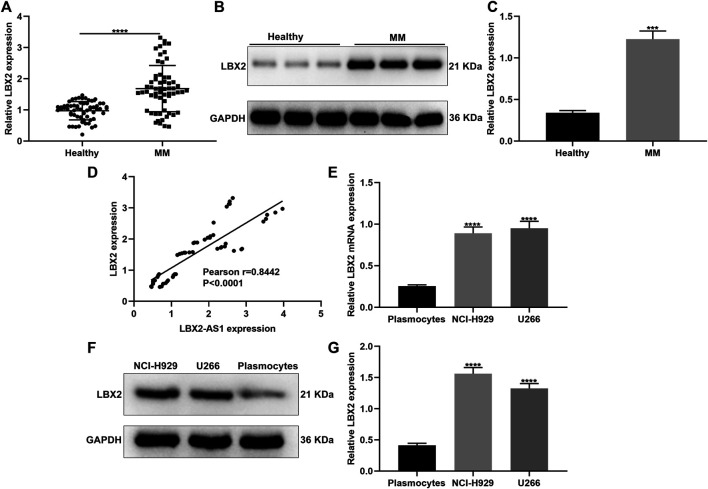
LBX2 is up-regulated in serum samples of MM and has a positive correlation to LBX2-AS1. **(A)** RT-qPCR for LBX2 mRNA expression in serum samples from 60 paired MM and healthy subjects. **(B, C)** LBX2 protein expression in serum samples from MM and healthy subjects. **(D)** Correlation between LBX2-AS1 and LBX2 expression in 60 MM patients. **(E)** The mRNA as well as **(F, G)** protein expression of LBX2-AS1 in CD138 + plasmocytes, NCI-H929 and U266 cells. ***p < 0.001; ****p < 0.0001.

This study also examined LBX2 expression in serum specimens from 60 paired MM and healthy subjects. Higher mRNA expression of LBX2 was found in MM patients compared to healthy participants (*p* < 0.0001; Figure 4A). Consistently, LBX2 protein displayed the elevated expression in MM patients in comparison to healthy controls (*p* < 0.001; Figures 4B,C). Correlation analysis confirmed that LBX2-AS1 expression exhibited a positive association with LBX2 expression in 60 cases of MM subjects (pearson r = 0.8442 and *p* < 0.0001; Figure 4D). LBX2 expression was also examined in CD138^+ ^plasmocytes as well as MM cells. Subsequently, LBX2 displayed the higher mRNA expression in NCI-H929 and U266 cells in comparison to plasmocytes (both *p* < 0.0001; Figure 4E). Similarly, LBX2 protein had the elevated expression levels in two MM cells than plasmocytes (both *p* < 0.0001; Figures 4F,G).

### Up-Regulation of LBX2-AS1 Enhances LBX2 Expression in MM Cells

**FIGURE 5:**
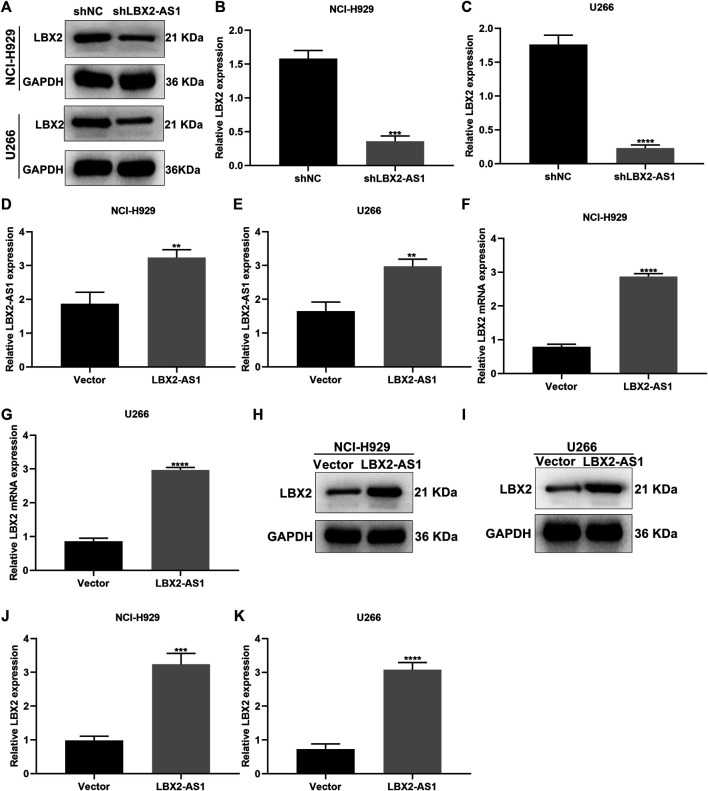
Up-regulation of LBX2-AS1 elevates LBX2 expression in MM cells. **(A–C)** Western blot for the expression of LBX2 protein in NCI-H929 as well as U266 cells under transfection by shNC or shLBX2-AS1. **(D, E)** The transfection effects of pcDNA3.1-LBX2-AS1 and empty vector in MM cells *via* RT-qPCR. **(F, G)** The expression of LBX2 mRNA in MM cells under transfection by empty vector and pcDNA3.1-LBX2-AS1 through RT-qPCR. **(H–K)** Western blot for the expression of LBX2 protein in MM cells under transfection by empty vector and pcDNA3.1-LBX2-AS1. **p < 0.01; ***p < 0.001; ****p < 0.0001.

We further analyzed whether LBX2-AS1 may alter LBX2 expression in MM cells. When transfected with shLBX2-AS1, this study examined LBX2 expression in MM cells *via* western blot (Figure 5A). Subsequently, shLBX2-AS1 transfection distinctly reduced the expression of LBX2 protein in NCI-H929 (*p* < 0.001; Figure 5B) and U266 cells (*p* < 0.0001; Figure 5C). The transfection effects of pcDNA3.1-LBX2-AS1 were verified in MM cells by RT-qPCR. As a result, LBX2-AS1 was overexpressed in NCI-H929 (Figure 5D) and U266 cells (Figure 5E; both *p* < 0.01) following transfection with pcDNA3.1-LBX2-AS1. Our RT-qPCR confirmed that pcDNA3.1-LBX2-AS1 distinctly elevated the expression of LBX2 mRNA in NCI-H929 (Figure 5F) as well as U266 cells (Figure 5G; both *p* < 0.0001). Its expression was also examined by western blot (Figures 5H,I). Consistently, higher expression of LBX2 protein was verified by pcDNA3.1-LBX2-AS1 transfection in NCI-H929 (Figure 5J; *p* < 0.001) and U266 cells (Figure 5K; *p* < 0.0001).

### LBX2-AS1 Enhances the mRNA Stability of LBX2 in MM Cells

**FIGURE 6:**
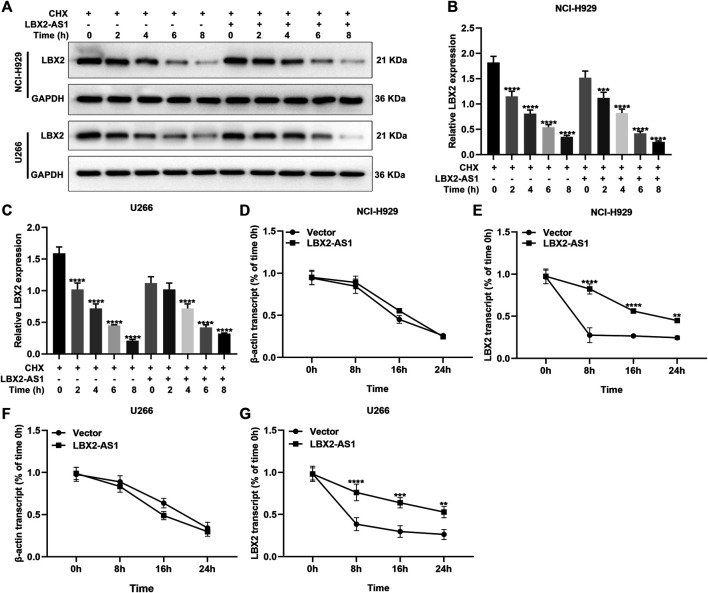
LBX2-AS1 enhances the mRNA stability of LBX2 in MM cells. **(A–C)** Western blot for the expression of LBX2 protein in pcDNA3.1-LBX2-AS1-transfected NCI-H929 and U266 cells after treatment with CHX for 0, 2, 4, 6, and 8 h. **(D–G)** RT-qPCR for the expression of LBX2 mRNA in pcDNA3.1-LBX2-AS1-transfected NCI-H929 and U266 cells following treatment with actinomycin D for 0, 8, 16, and 24 h. **p < 0.01; ***p < 0.001; ****p < 0.0001.

Antisense lncRNAs may exert a regulatory role on target genes through binding to mRNAs (Zhao et al., 2018). Here, MM cells with pcDNA3.1-LBX2-AS1 transfection were treated with CHX for 0, 2, 4, 6, and 8 h. Western blot was presented to examine LBX2 expression. As shown in Figures 6A–C, pcDNA3.1-LBX2-AS1 did not change the protein stability of LBX2. Moreover, after treatment with actinomycin D in pcDNA3.1-LBX2-AS1-transfected MM cells, we examined the expression of LBX2 mRNA by RT-qPCR. As a result, pcDNA3.1-LBX2-AS1 could enhance the mRNA stability of LBX2 both in NCI-H929 (Figures 6D,E) and U266 cells (Figures 6F,G).

### LBX2-AS1 Facilitates MM Progression Through Enhancing mRNA Stability of LBX2

**FIGURE 7:**
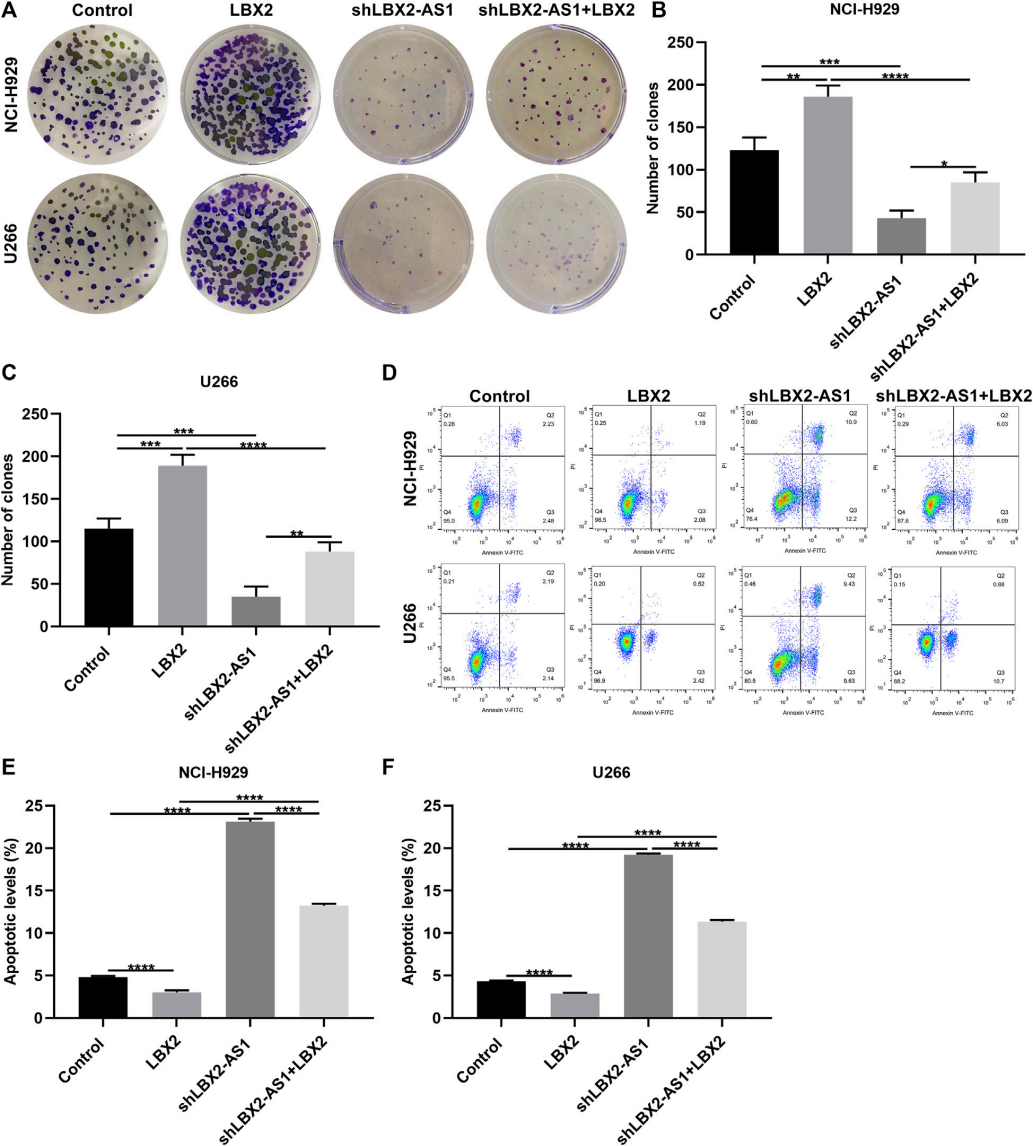
LBX2-AS1 enhances proliferative ability and decreases apoptotic levels in MM cells through enhancing mRNA stability of LBX2. **(A–C)** The number of clones of NCI-H929 and U266 cells under transfection by pcDNA3.1-LBX2 and/or shLBX2-AS1. **(D–F)** Flow cytometry for the apoptotic rates of MM cells under transfection by pcDNA3.1-LBX2 and/or shLBX2-AS1. *p < 0.05; **p < 0.01; ***p < 0.001; ****p < 0.0001.

Clone formation assay demonstrated that pcDNA3.1-LBX2 distinctly elevated the number of clones in NCI-H929 (*p* < 0.01) as well as U266 cells (*p* < 0.001; Figures 7A–C). Meanwhile, the number of clones was markedly reduced by shLBX2-AS1. Moreover, LBX2-AS1 knockdown reversed the increase in the number of clones induced by pcDNA3.1-LBX2 in NCI-H929 as well as U266 cells (both *p* < 0.0001). Apoptosis was evaluated by flow cytometry. As a result, lowered apoptotic levels were found in two MM cells by pcDNA3.1-LBX2 transfection compared to controls (both *p* < 0.0001; Figures 7D–F). On the contrary, shLBX2-AS1 facilitated the apoptosis of NCI-H929 and U266 cells (both *p* < 0.0001). Moreover, LBX2-AS1 knockdown ameliorated the enhancement in apoptosis induced by LBX2 overexpression in MM cells. Collectively, LBX2-AS1 may induce MM progression through enhancing mRNA stability of LBX2.

### Silencing LBX2-AS1 Suppresses Tumor Growth of MM

**FIGURE 8:**
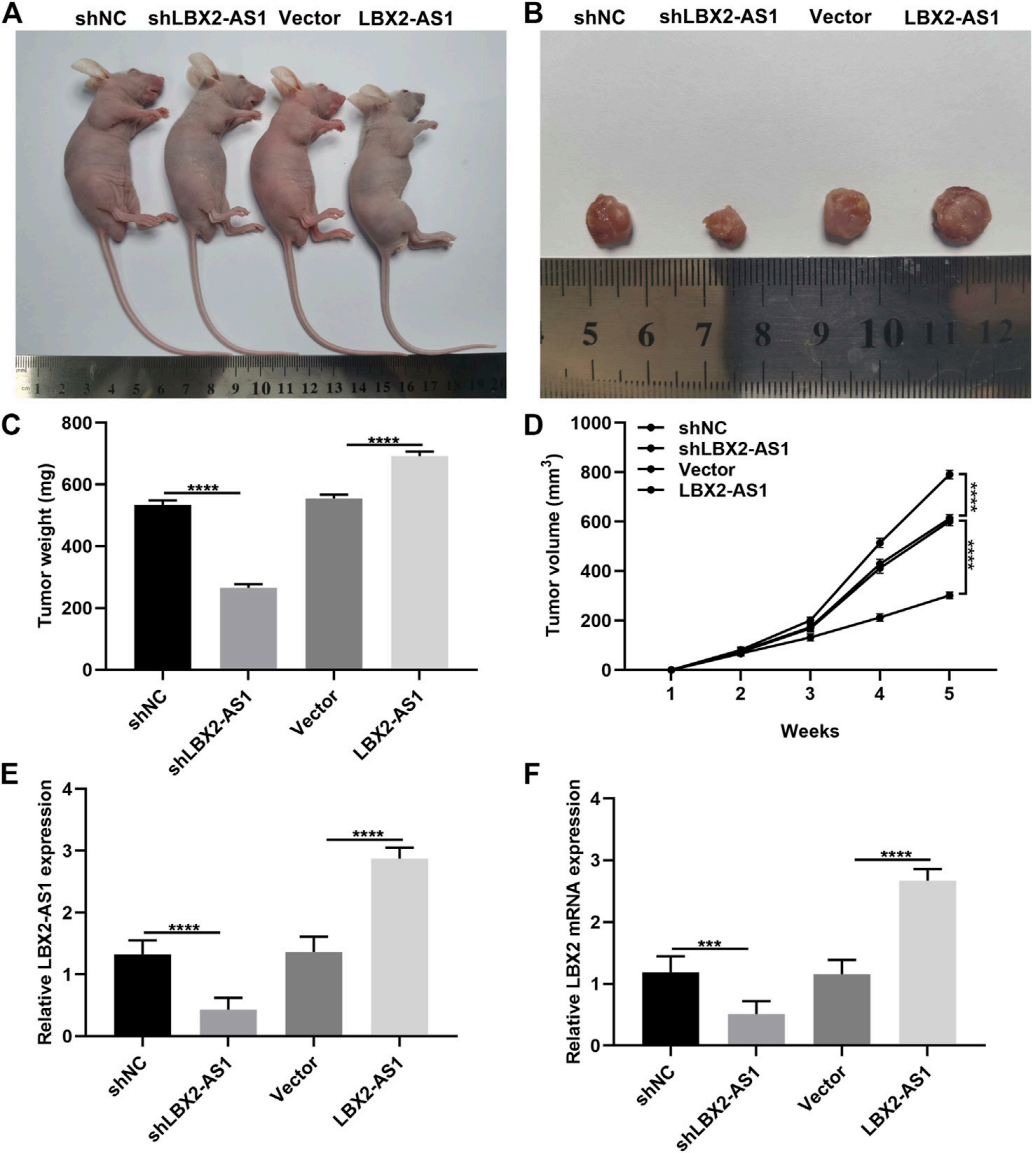
Silencing LBX2-AS1 suppresses tumor growth of MM. **(A)** Representative images of nude mice treated with NCI-H929 cells that were transfected with shNC, shLBX2-AS1, empty vector and LBX2-AS1 overexpression. **(B)** Representative images of tumors from above nude mice after 5 weeks. **(C)** Detection of tumor weight after treatment for 5 weeks. **(D)** Detection of tumor volume in each week. **(E, F)** RT-qPCR examining the expression of LBX2-AS1 and LBX2 in tumor tissues from above nude mice after treatment for 5 weeks. ***p < 0.001; ****p < 0.0001.

To investigate the function of LBX2-AS1 on MM progression, we further established xenograft tumor models. Our data showed that LBX2-AS1 knockdown distinctly suppressed tumor growth and the opposite results were observed when LBX2-AS1 was overexpressed (Figures 8A–D). The expression of LBX2-AS1 and LBX2 was examined in tumor tissues *via* RT-qPCR. As a result, LBX2-AS1 expression was substantially reduced by shLBX2-AS1, while its expression was enhanced by LBX2-AS1 overexpression plasmid (Figure 8E). Moreover, we found that LBX2-AS1 knockdown weakened the expression of LBX2 and the opposite results were investigated when LBX2-AS1 overexpression in MM tumor tissues (Figure 8F).

## Discussion

MM is a malignancy with complex and unstable genomic alterations (Hu et al., 2020). Multiple lncRNAs with oncogenic or tumor-suppressive roles are involved in MM initiation and progression, demonstrating that lncRNAs may be efficient diagnostic or prognostic markers as well as promising therapeutic targets in MM. For example, MALAT1 may facilitate tumorigenesis, invasion as well as glycolysis in MM through miR-1271-5p/SOX13 pathway (Liu et al., 2020). Here, this study proposed that LBX2-AS1 as a diagnostic marker was up-regulated in serum samples from MM patients. It may induce MM progression by enhancing mRNA stability of LBX2. Thus, LBX2-AS1 could be a novel promising therapeutic target against MM.

This study recruited 60 paired MM patients and healthy participants. LBX2-AS1 up-regulation was confirmed in MM serum samples. As previous studies, up-regulated LBX2-AS1 has been found in gastric cancer (Yang et al., 2020), ovarian cancer (Cao et al., 2021), esophageal squamous cell carcinoma (Zhang et al., 2019) as well as hepatocellular carcinoma (Wang et al., 2020c). There is a huge need to discover non-invasive biomarkers with high specificity in MM (Chi et al., 2019). LncRNAs with cell-, tissue or tumor-specific expression have emerged as convenient as well as less invasive diagnostic biomarkers (Chandra Gupta and Nandan Tripathi, 2017). Moreover, lncRNAs are stably expressed in blood and other body fluids, so they are suitable biomarkers for disease diagnosis. Our ROC curves demonstrated that circulating LBX2-AS1 was a sensitive diagnostic marker of MM. However, its diagnostic potential should be verified in a larger MM cohort. The overexpression of LBX2-AS1 was further validated in MM cells. Our results confirmed that LBX2-AS1 expression was markedly increased in two MM cells than normal human marrow CD138^+ ^plasmocytes. Based on the up-regulation of LBX2-AS1 both in MM patients and cells, we inferred that LBX2-AS1 might participate in MM progression. Also, it was mainly expressed in cytoplasm of MM cells, indicating that it primarily exerted a regulatory function at the post-transcriptional level. Alterations in lncRNA expression facilitate cancer progression by facilitating proliferation and restraining apoptosis (Bhan et al., 2017). It is of significance to develop lncRNA-based therapeutics against cancers (Choudhari et al., 2020). In the case of stable or remission of MM, it is key to effectively inhibit proliferation and induce apoptosis in MM cells, thereby removing the small residual MM cells and prolonging the survival duration of MM patients (Barwick et al., 2019). Here, targeting LBX2-AS1 may weaken proliferative ability and tumor growth as well as elevate apoptotic levels in MM cells.

LBX2 was overexpressed in serum specimens from MM subjects and displayed a positive correlation to LBX2-AS1. LBX2-AS1 up-regulation could markedly enhance LBX2 expression in MM cells. About 70% of the genes have antisense lncRNAs (He et al., 2008). Antisense lncRNAs are in relation to the expression of their sense strand genes, suggesting that they may widely participate in regulating the expression of protein-coding genes. It has been confirmed that antisense lncRNAs may control cancer progression by affecting mRNA stability of oncogenes (Jadaliha et al., 2018). For instance, EGFR-AS1 facilitates tumor growth as well as metastases through altering mRNA stability of EGFR in renal carcinoma (Wang et al., 2019). LDLRAD4-AS1 induces metastases through disrupting mRNA stability of LDLRAD4 in colorectal cancer (Mo et al., 2020). FOXC2-AS1 may enhance FOXC2 mRNA stability, thereby promoting colorectal cancer development by activating Ca^2+^-FAK pathway (Pan and Xie, 2020). TTN-AS1 enhances tumorigenesis as well as metastases *via* building up TTN expression in skin cutaneous melanoma (Wang et al., 2020b). DDX11-AS1 accelerates proliferation, invasion as well as metastasis in osteosarcoma through stabilizing DDX11 (Zhang et al., 2020). Also, HHIP-AS1 restrains hepatocellular carcinoma development by stabilizing HHIP mRNA (Bo et al., 2019). Here, LBX2-AS1 overexpression may strengthen mRNA stability of LBX2 in MM cells. Our rescue experiments demonstrated that LBX2-AS1 could induce MM progression by elevating mRNA stability of LBX2.

## Conclusion

Collectively, LBX2-AS1 was up-regulated in MM serum specimens and an underlying diagnostic marker of MM. Targeting LBX2-AS1 may weaken MM progression by heightening mRNA stability of LBX2. More studies are required to verify the clinical implication as well as biological role of LBX2-AS1 in MM.

## Data Availability

The original contributions presented in the study are included in the article/Supplementary Material, further inquiries can be directed to the corresponding author.

## Abbreviations

AUC: area under the curve; CCK-8: cell counting kit-8; CHX: cycloheximide; MM: multiple myeloma; lncRNA: long non-coding RNA; PCR PI: propidium iodide; ROC: receiver operating characteristic curve; RT-qPCR: Real-time quantitative; shNC: shRNA negative control.
