# Supplementary figures and images for: LBX2-AS1 as a Novel Diagnostic Biomarker and Therapeutic Target Facilitates Multiple Myeloma Progression by Enhancing mRNA Stability of LBX2
Source: Front Mol Biosci. 2021 Sep 6;8:706570. doi: 10.3389/fmolb.2021.706570 (PMC8450339; doi:10.3389/fmolb.2021.706570)

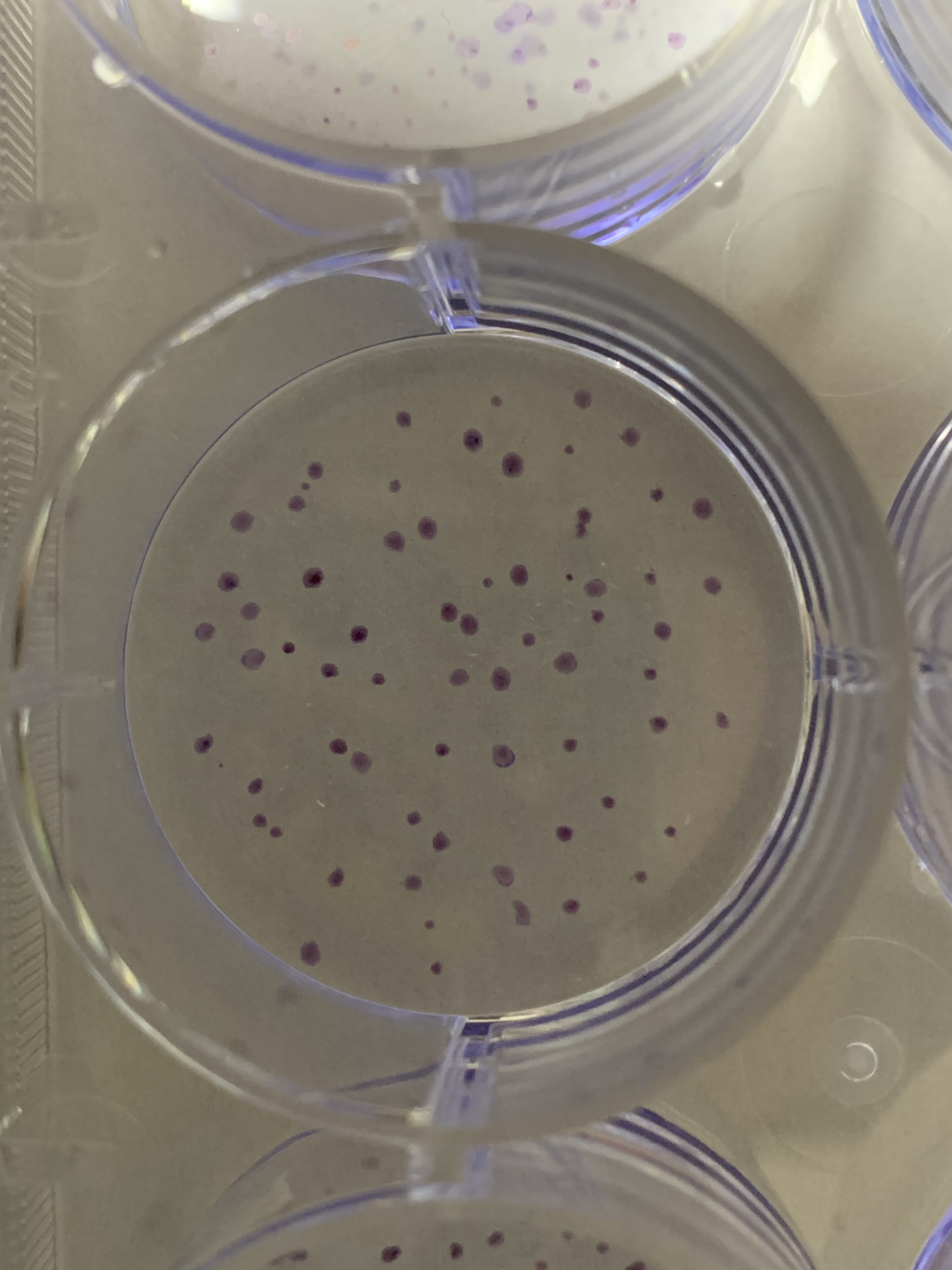

Supplement: Supplementary file 1 [file DataSheet1.ZIP › Original Source Data-Figure 1-4/Figure 2/Figure 2E/shLBX2-NCI-H929.JPG]

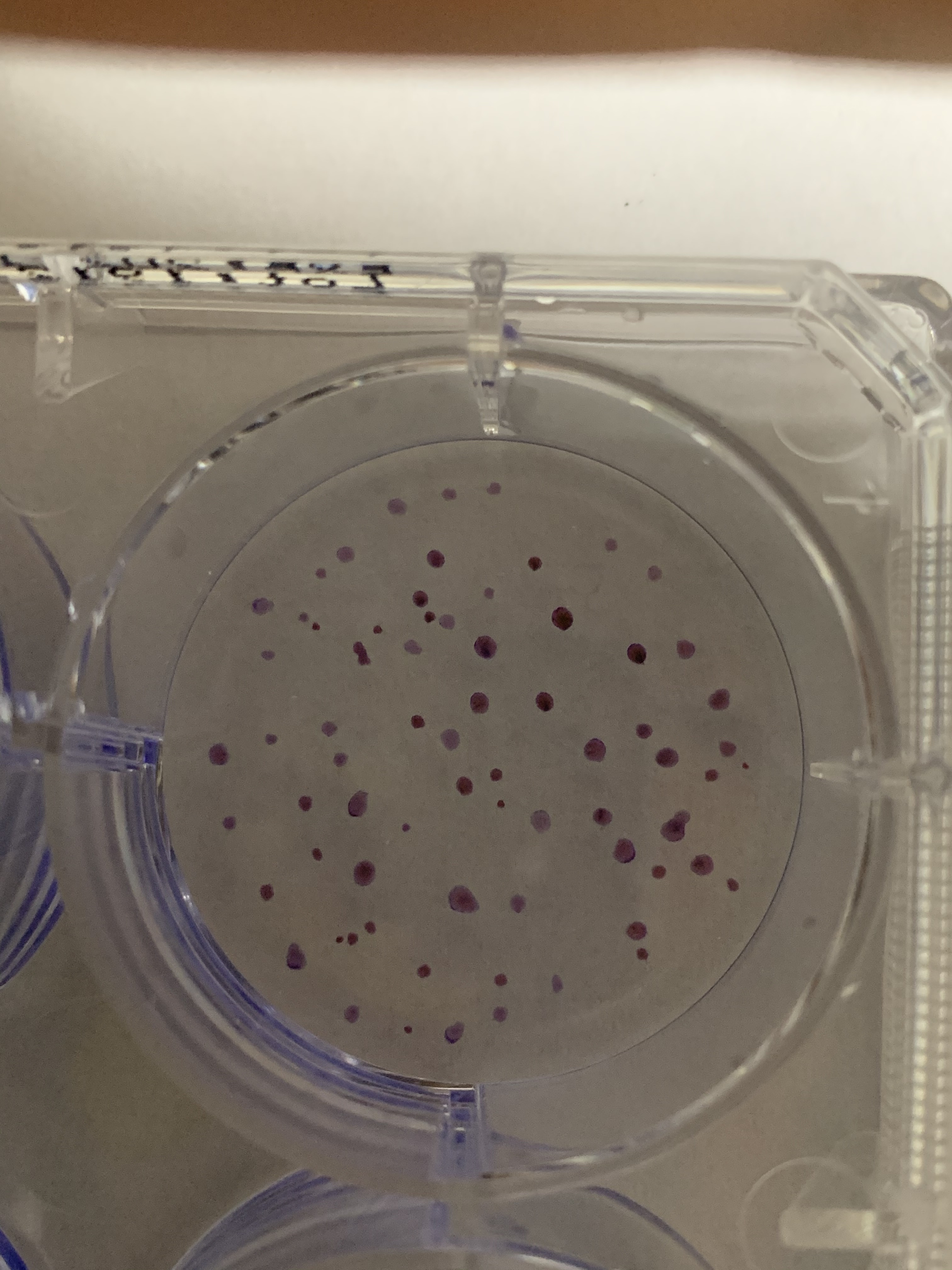

Supplement: Supplementary file 1 [file DataSheet1.ZIP › Original Source Data-Figure 1-4/Figure 2/Figure 2E/shLBX2-U266.JPG]

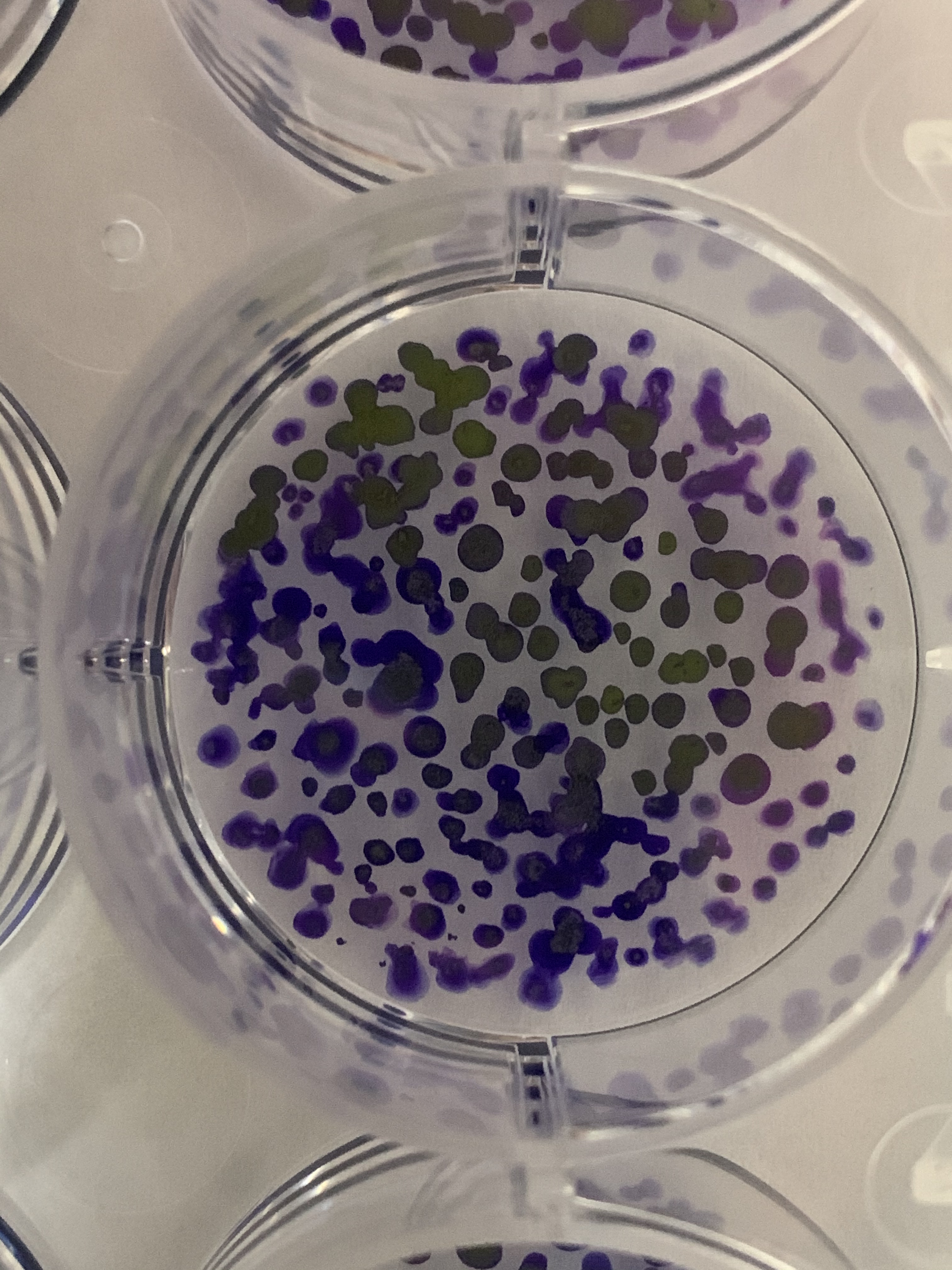

Supplement: Supplementary file 1 [file DataSheet1.ZIP › Original Source Data-Figure 1-4/Figure 2/Figure 2E/shNC-NCI-H929.JPG]

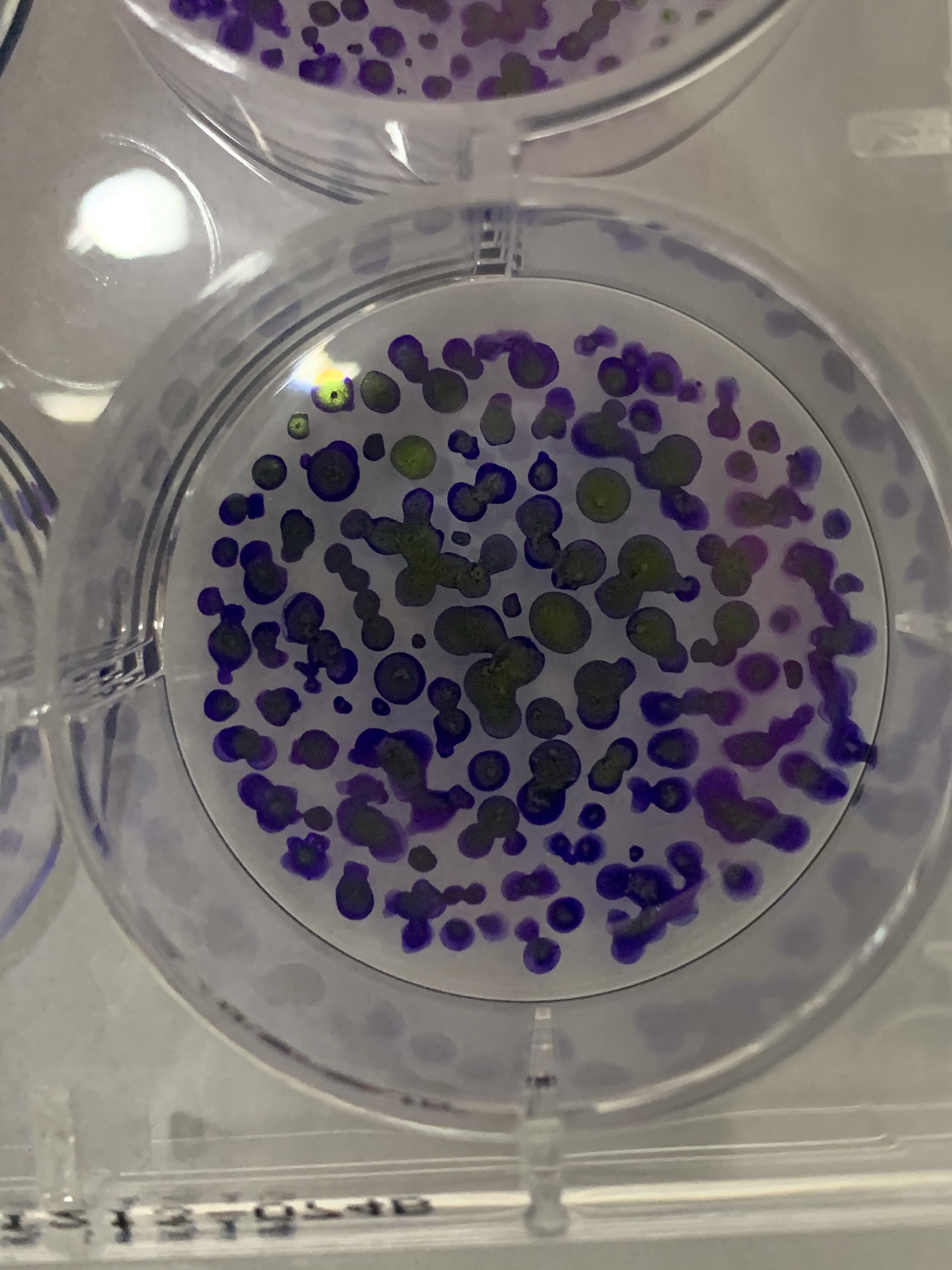

Supplement: Supplementary file 1 [file DataSheet1.ZIP › Original Source Data-Figure 1-4/Figure 2/Figure 2E/shNC-U266.JPG]

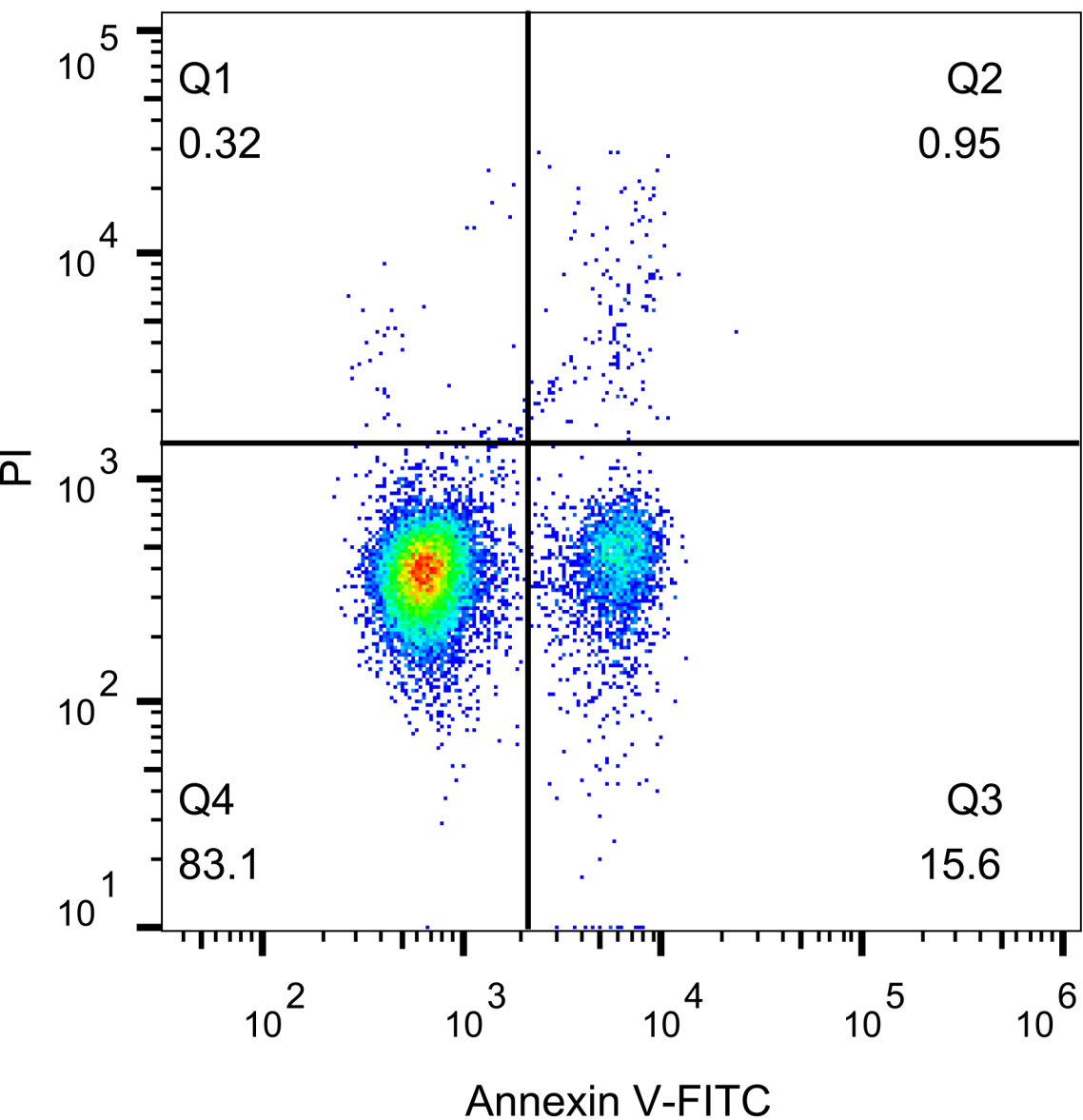

15-1.fcs

Single Cells

15629

Supplement: Supplementary file 1 [file DataSheet1.ZIP › Original Source Data-Figure 1-4/Figure 3/Figure 3A/NCI-H929-shLBX2-AS1.pdf]

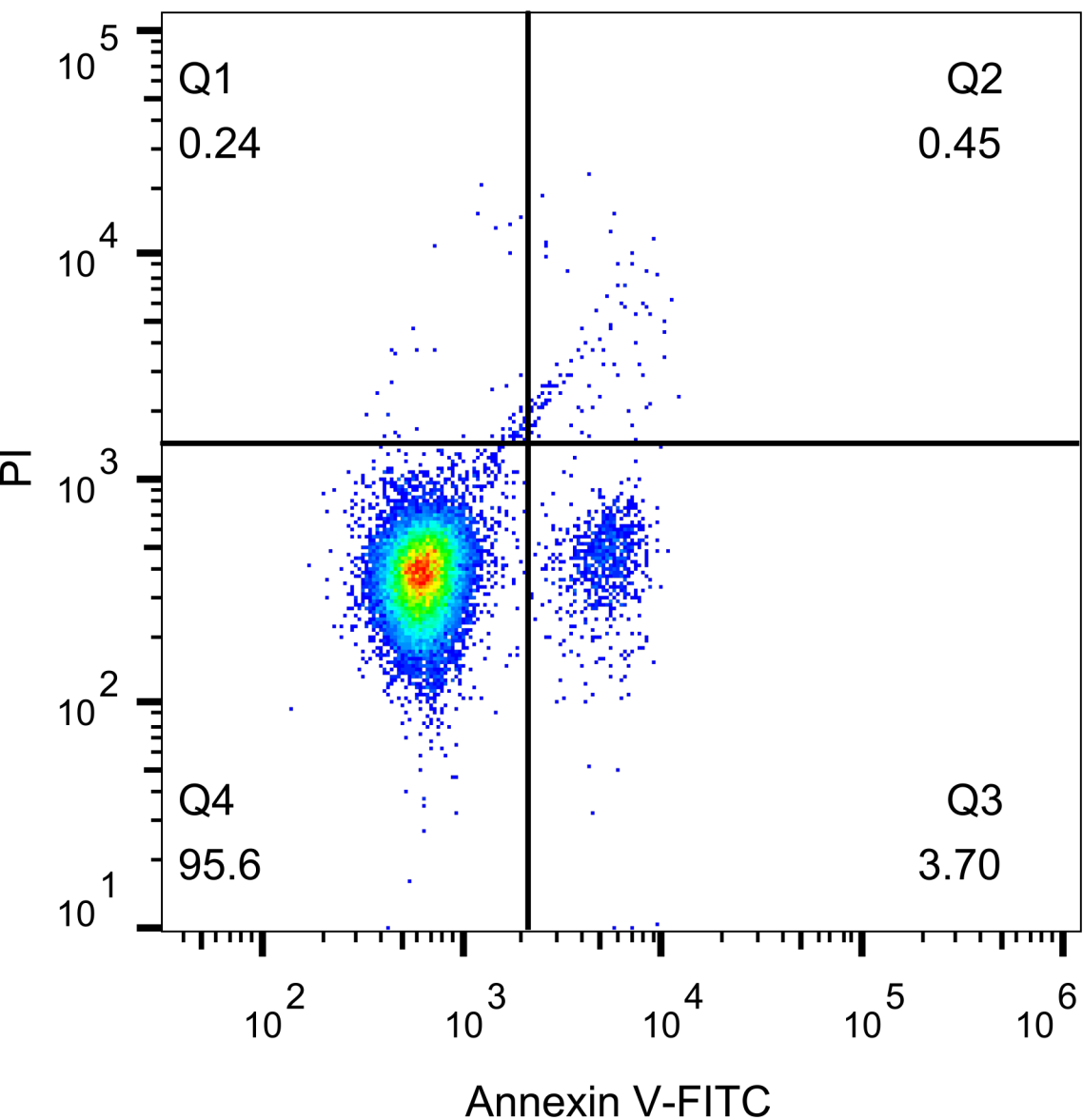

3-1.fcs

Single Cells

20671

Supplement: Supplementary file 1 [file DataSheet1.ZIP › Original Source Data-Figure 1-4/Figure 3/Figure 3A/NCI-H929-shNC.pdf]

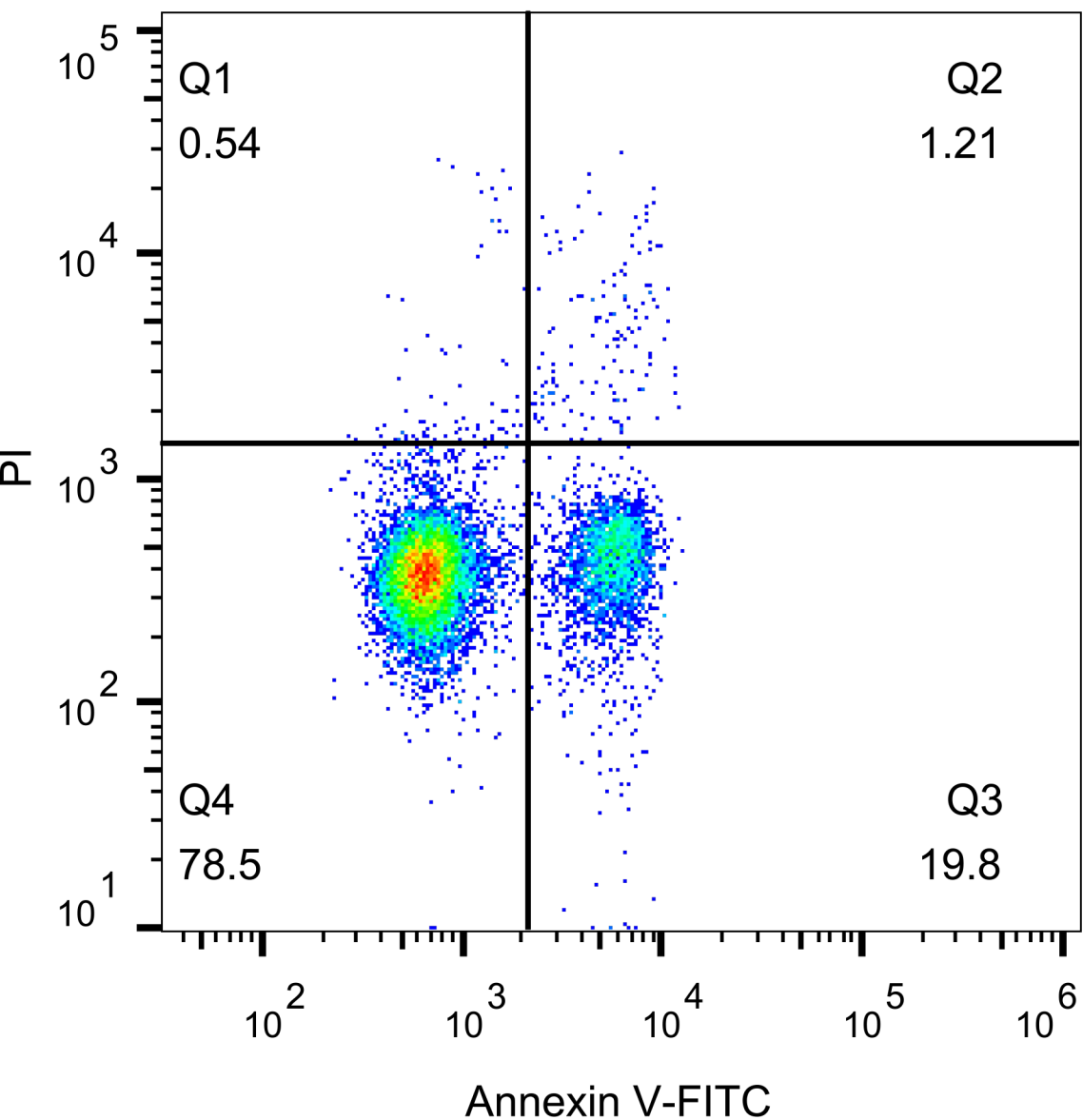

20-1.fcs

Single Cells

12330

Supplement: Supplementary file 1 [file DataSheet1.ZIP › Original Source Data-Figure 1-4/Figure 3/Figure 3A/U266-shLBX2-AS1.pdf]

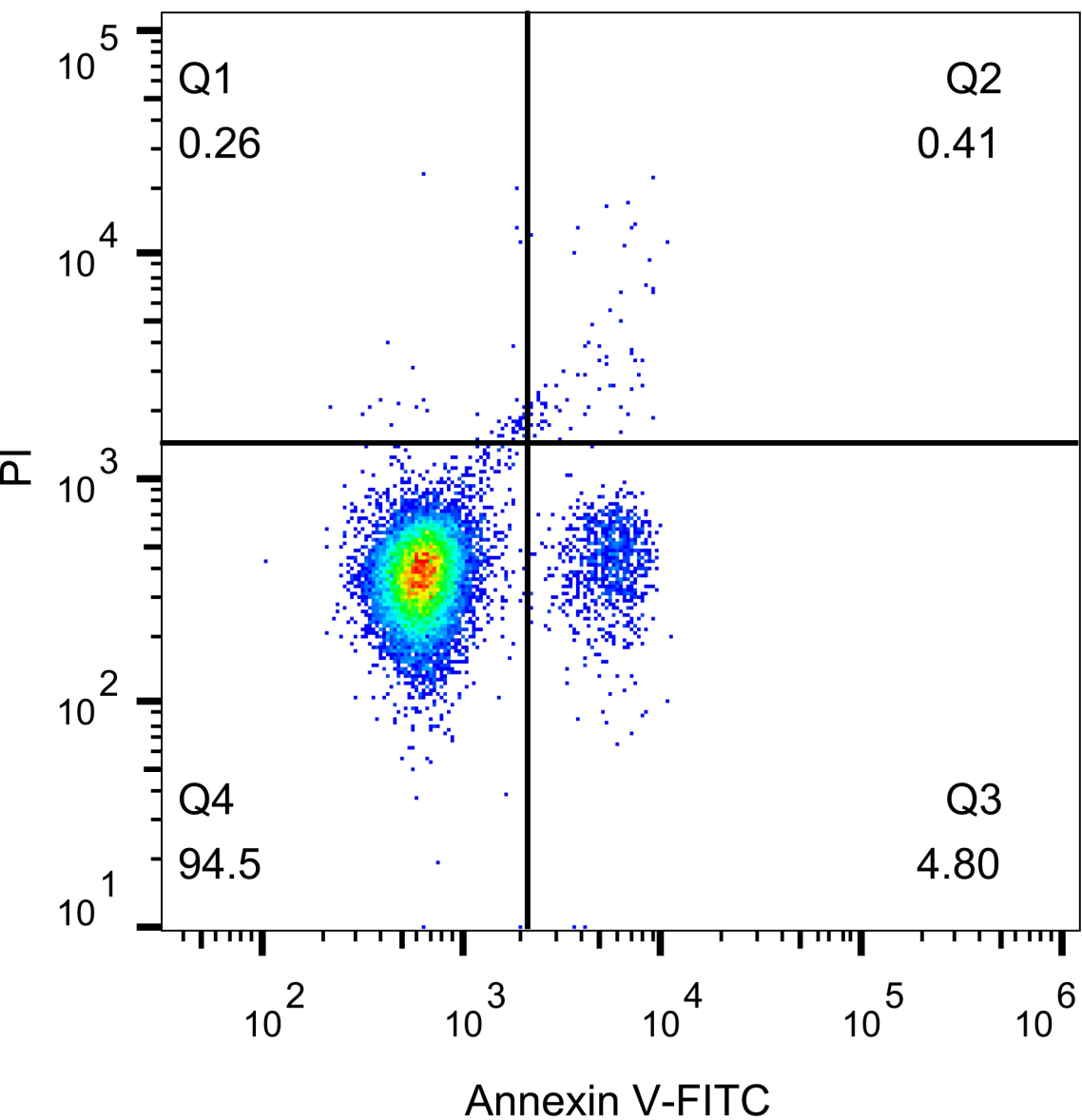

4-1.fcs

Single Cells

17173

Supplement: Supplementary file 1 [file DataSheet1.ZIP › Original Source Data-Figure 1-4/Figure 3/Figure 3A/U266-shNC.pdf]

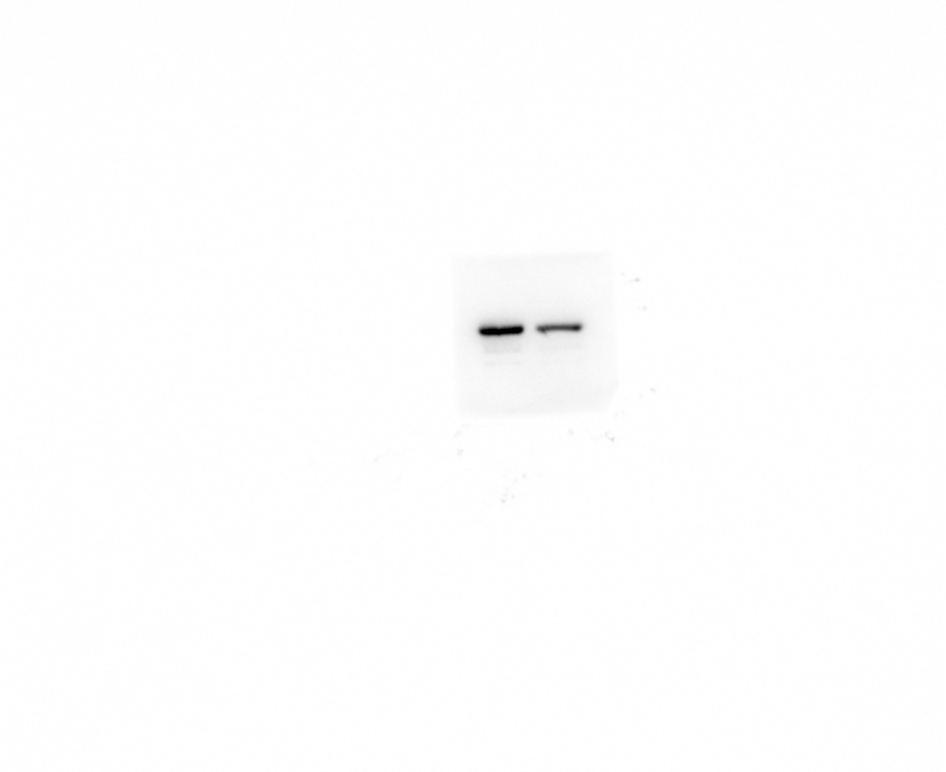

Supplement: Supplementary file 1 [file DataSheet1.ZIP › Original Source Data-Figure 1-4/Figure 3/Figure 3D/Bax-NCI-H929.tif]

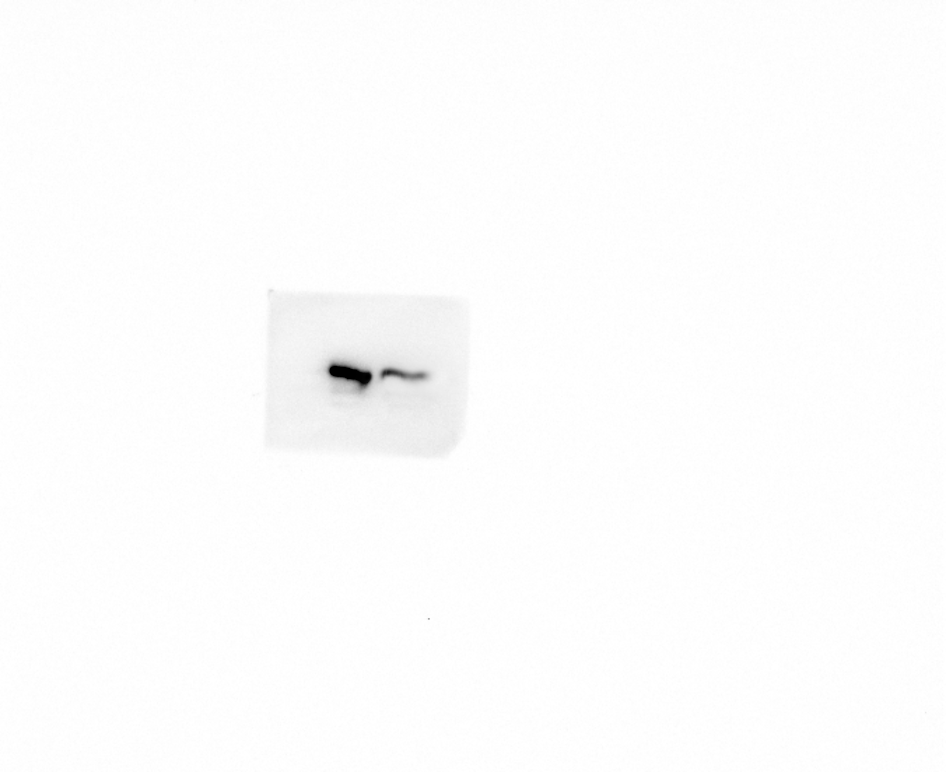

Supplement: Supplementary file 1 [file DataSheet1.ZIP › Original Source Data-Figure 1-4/Figure 3/Figure 3D/Bax-U266.tif]

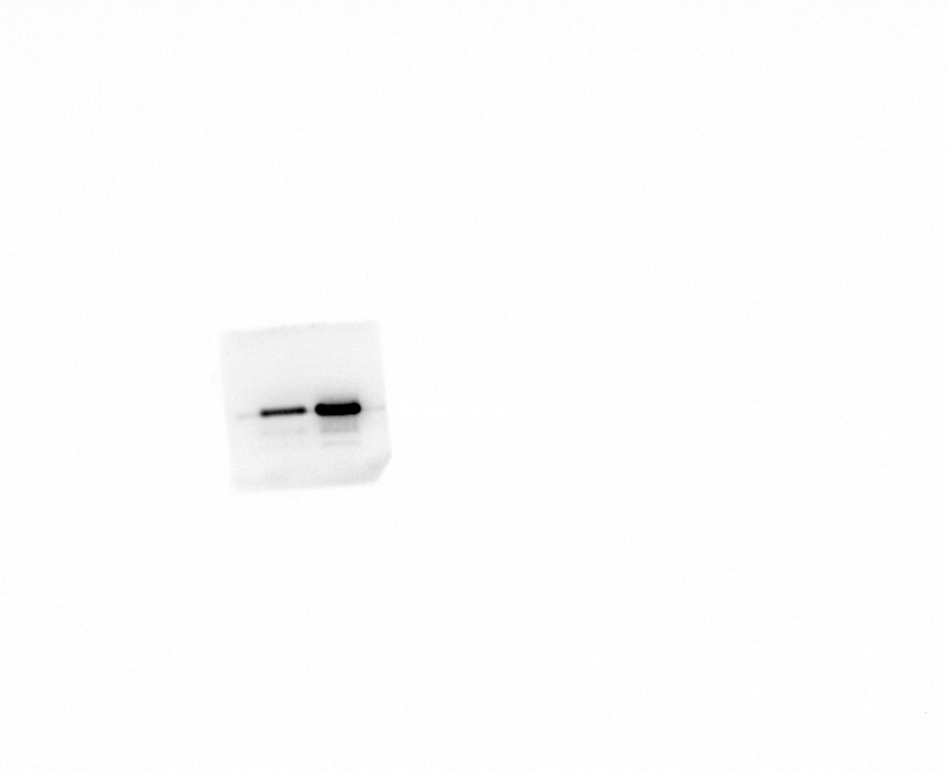

Supplement: Supplementary file 1 [file DataSheet1.ZIP › Original Source Data-Figure 1-4/Figure 3/Figure 3D/Bcl-2-NCI-H929.tif]

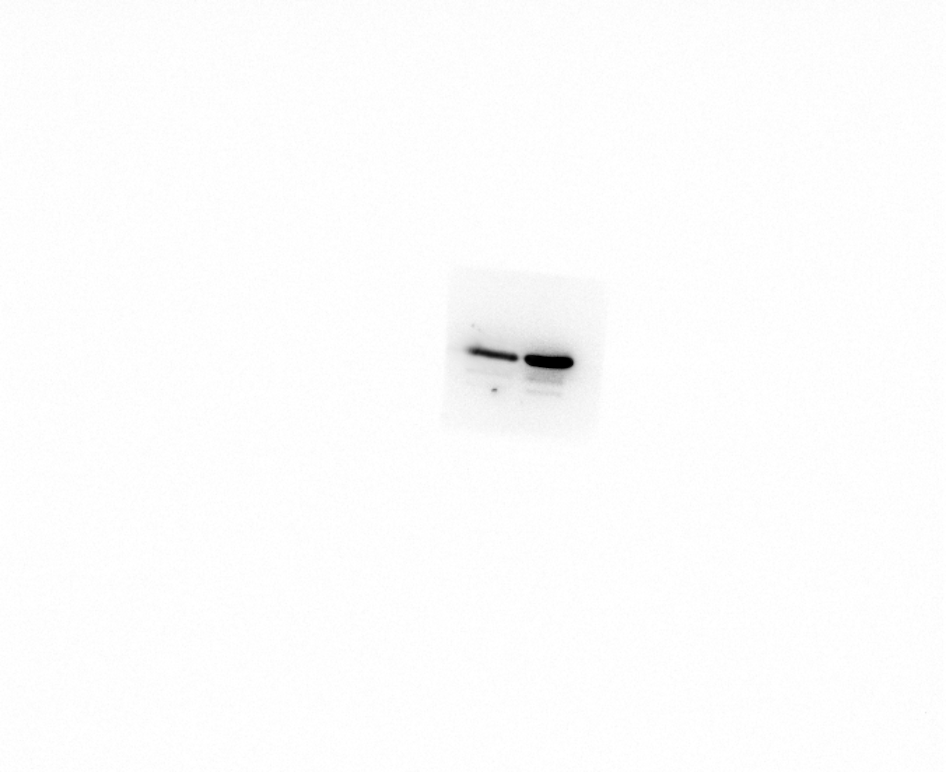

Supplement: Supplementary file 1 [file DataSheet1.ZIP › Original Source Data-Figure 1-4/Figure 3/Figure 3D/Bcl-2-U266.tif]

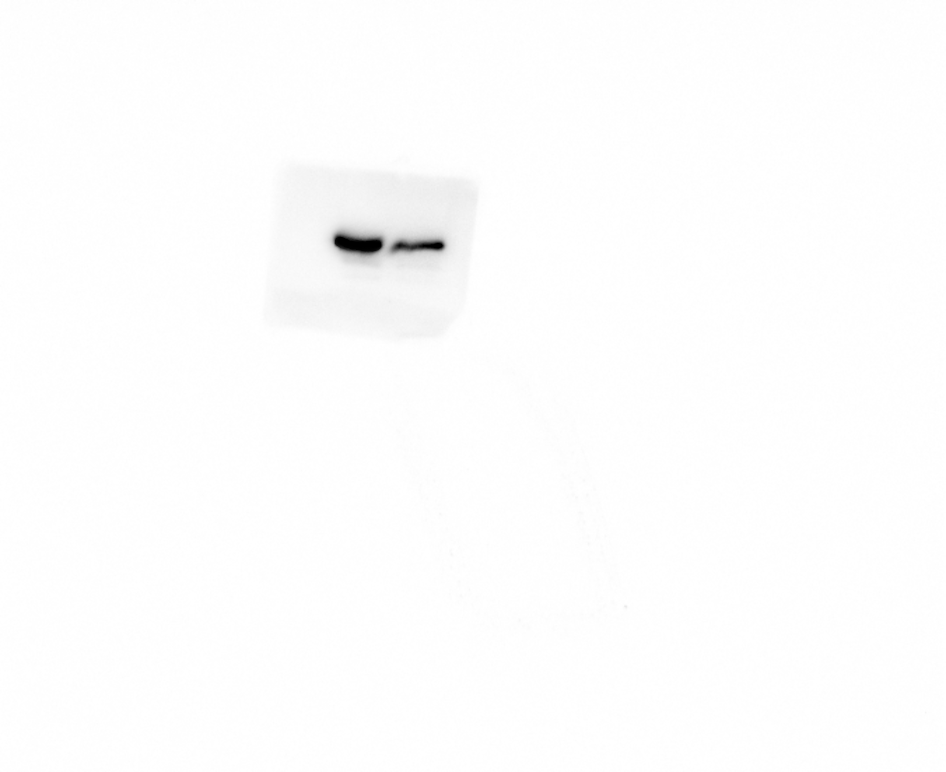

Supplement: Supplementary file 1 [file DataSheet1.ZIP › Original Source Data-Figure 1-4/Figure 3/Figure 3D/Cleaved caspase3-NCI-H929.tif]

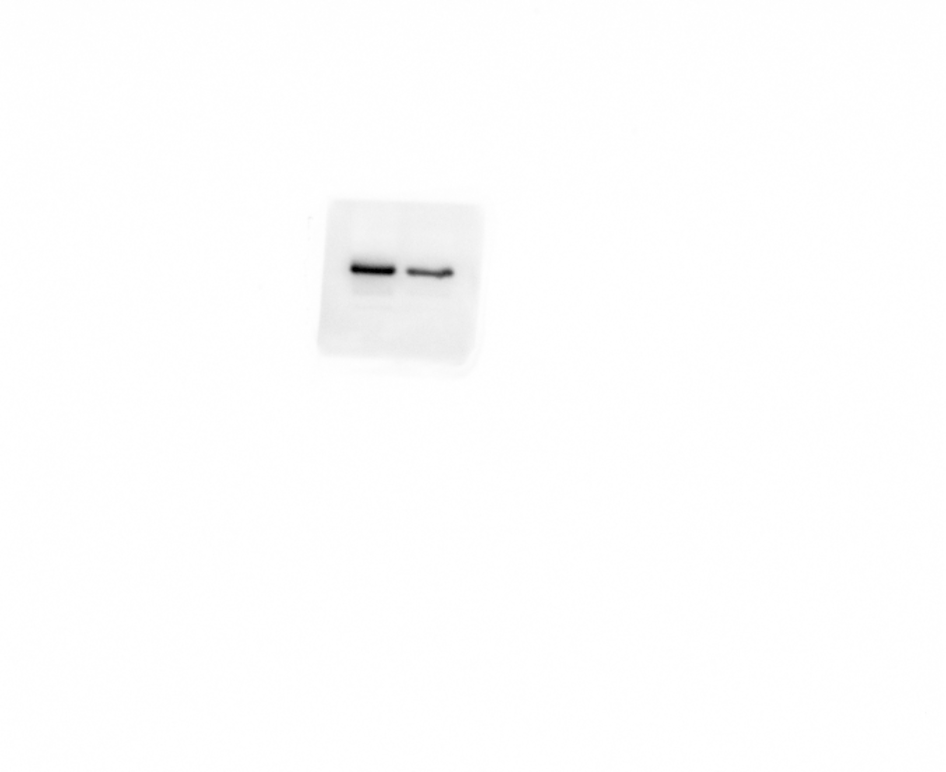

Supplement: Supplementary file 1 [file DataSheet1.ZIP › Original Source Data-Figure 1-4/Figure 3/Figure 3D/Cleaved caspase3-U266.tif]

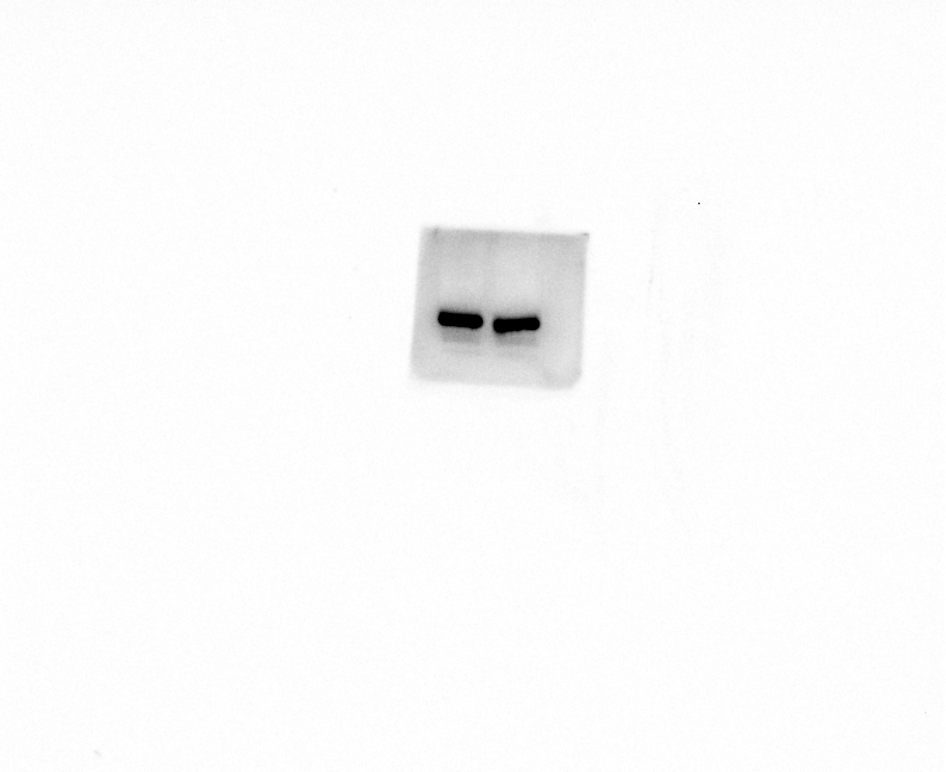

Supplement: Supplementary file 1 [file DataSheet1.ZIP › Original Source Data-Figure 1-4/Figure 3/Figure 3D/GAPDH-NCI-H929.tif]

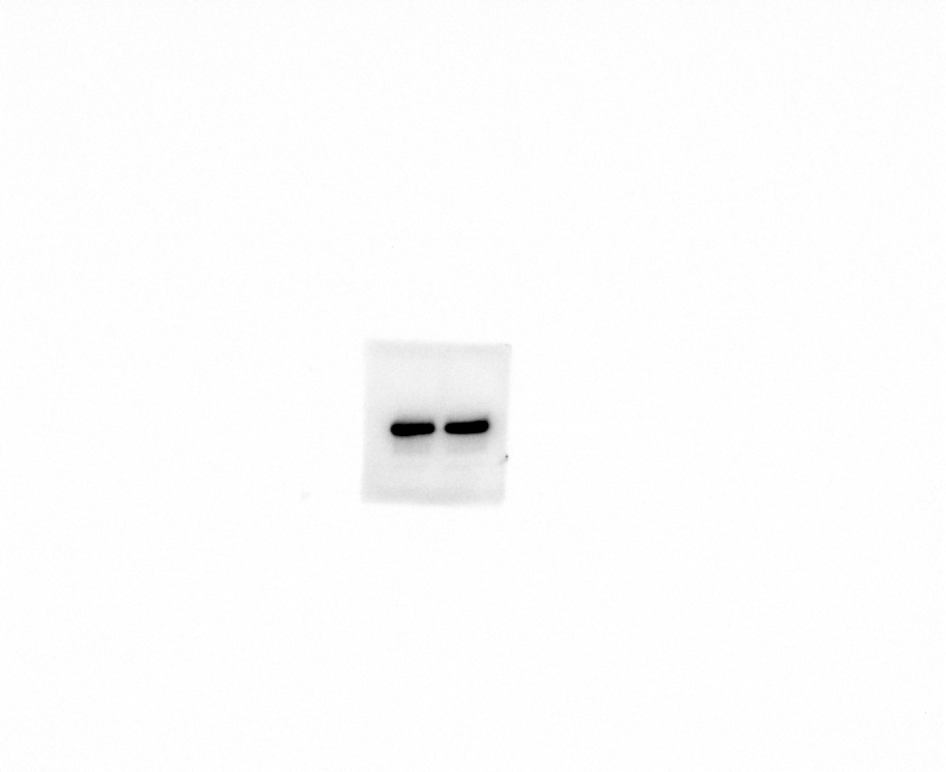

Supplement: Supplementary file 1 [file DataSheet1.ZIP › Original Source Data-Figure 1-4/Figure 3/Figure 3D/GAPDH-U266.tif]

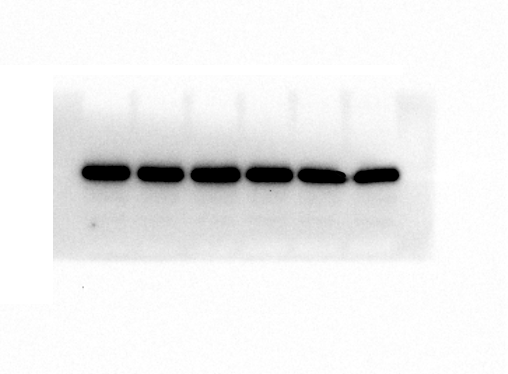

Supplement: Supplementary file 1 [file DataSheet1.ZIP › Original Source Data-Figure 1-4/Figure 4/Figure 4B/GAPDH.tif]

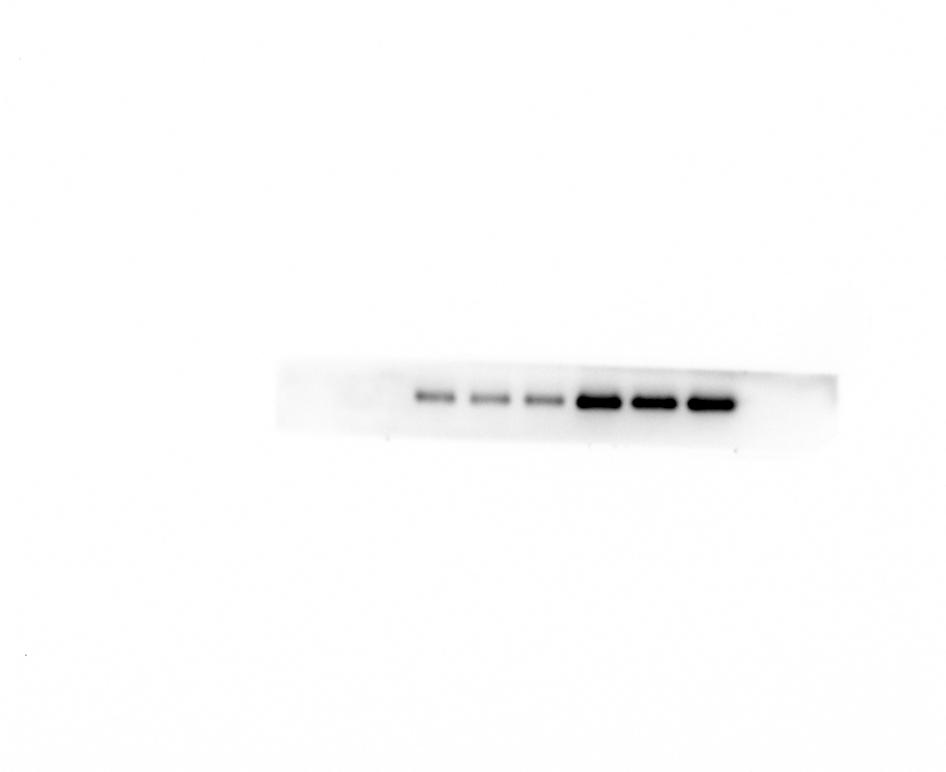

Supplement: Supplementary file 1 [file DataSheet1.ZIP › Original Source Data-Figure 1-4/Figure 4/Figure 4B/LBX2.tif]

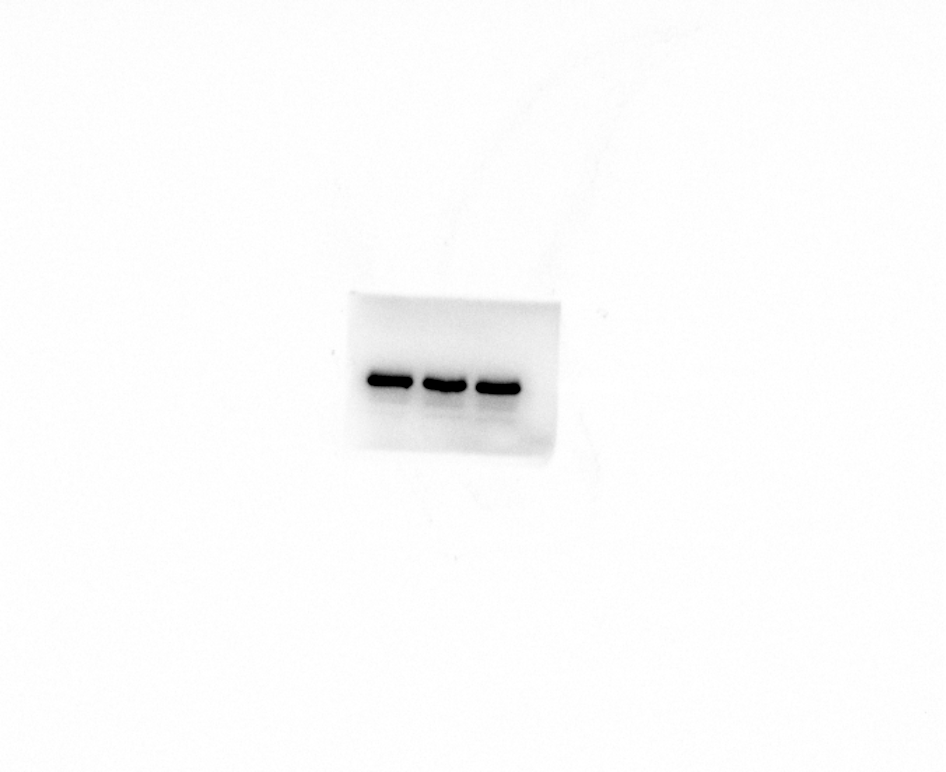

Supplement: Supplementary file 1 [file DataSheet1.ZIP › Original Source Data-Figure 1-4/Figure 4/Figure 4F/GAPDH.tif]

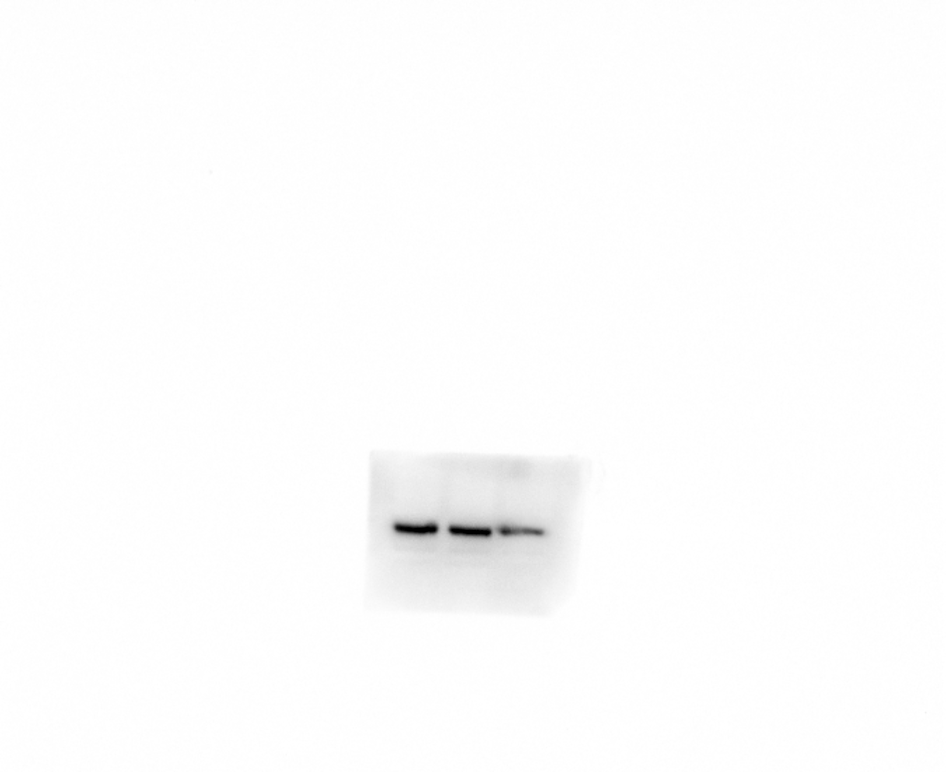

Supplement: Supplementary file 1 [file DataSheet1.ZIP › Original Source Data-Figure 1-4/Figure 4/Figure 4F/LBX2.tif]

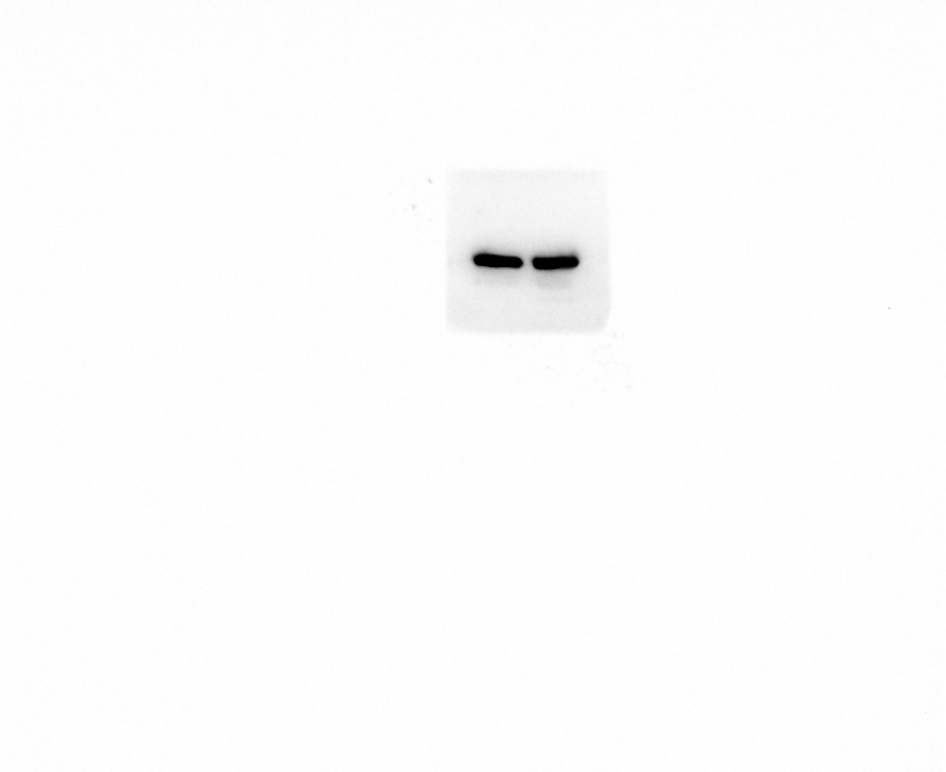

Supplement: Supplementary file 2 [file DataSheet2.ZIP › Original Source Data-Figure 5-7/Figure 5/Figure 5A/GAPDH-NCI-H929.tif]

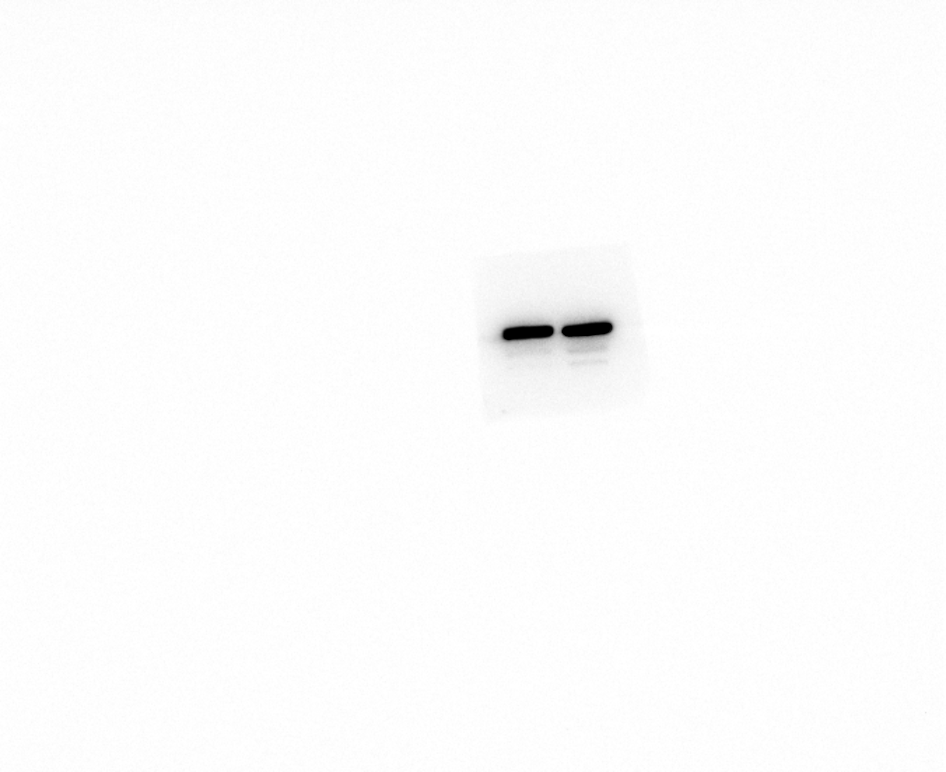

Supplement: Supplementary file 2 [file DataSheet2.ZIP › Original Source Data-Figure 5-7/Figure 5/Figure 5A/GAPDH-U266.tif]

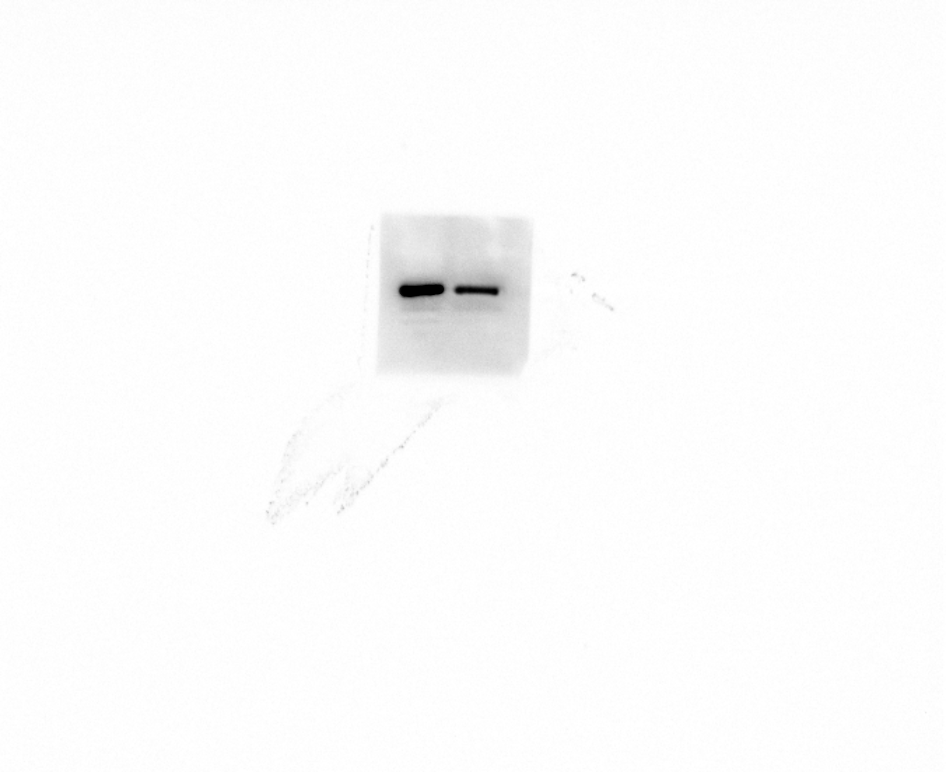

Supplement: Supplementary file 2 [file DataSheet2.ZIP › Original Source Data-Figure 5-7/Figure 5/Figure 5A/LBX2-NCI-H929.tif]

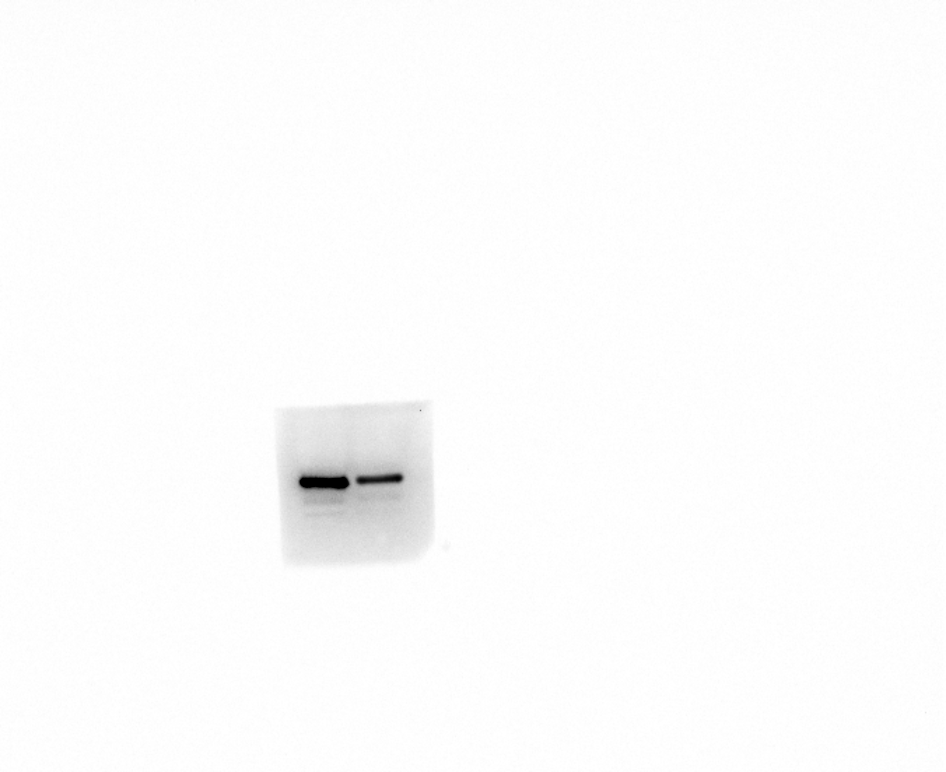

Supplement: Supplementary file 2 [file DataSheet2.ZIP › Original Source Data-Figure 5-7/Figure 5/Figure 5A/LBX2-U266.tif]

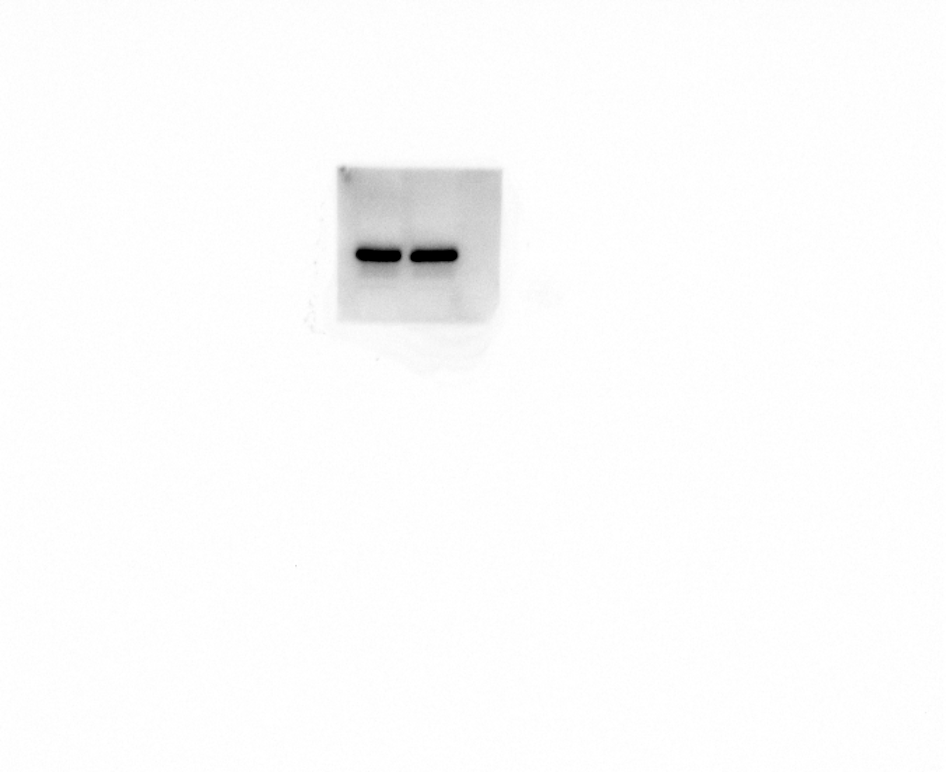

Supplement: Supplementary file 2 [file DataSheet2.ZIP › Original Source Data-Figure 5-7/Figure 5/Figure 5H/GAPDH.tif]

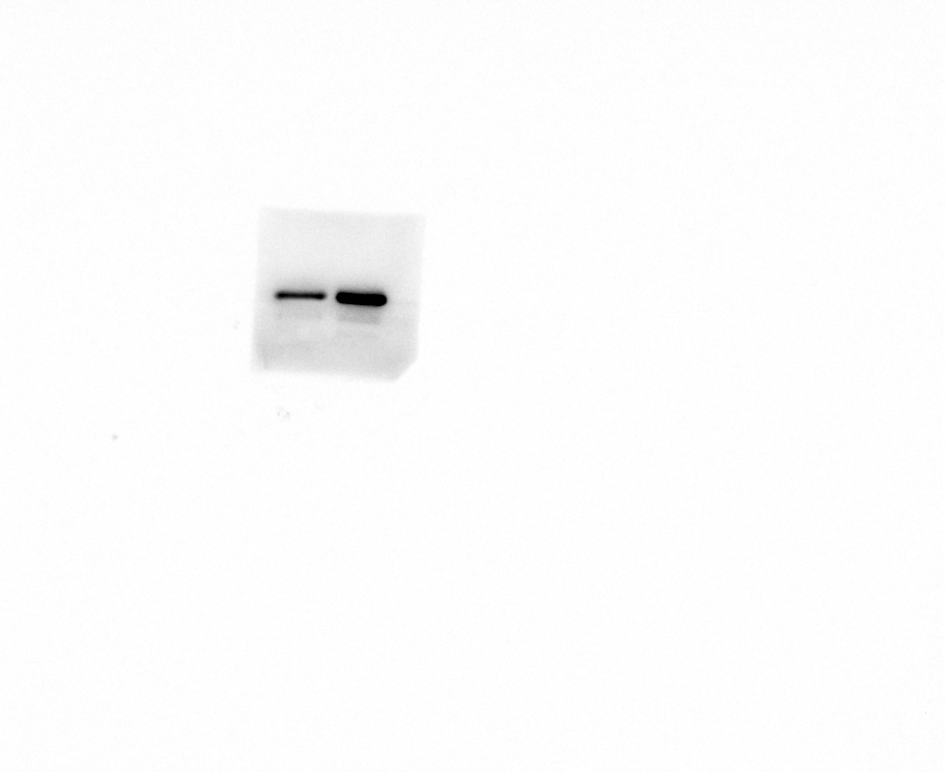

Supplement: Supplementary file 2 [file DataSheet2.ZIP › Original Source Data-Figure 5-7/Figure 5/Figure 5H/LBX2.tif]

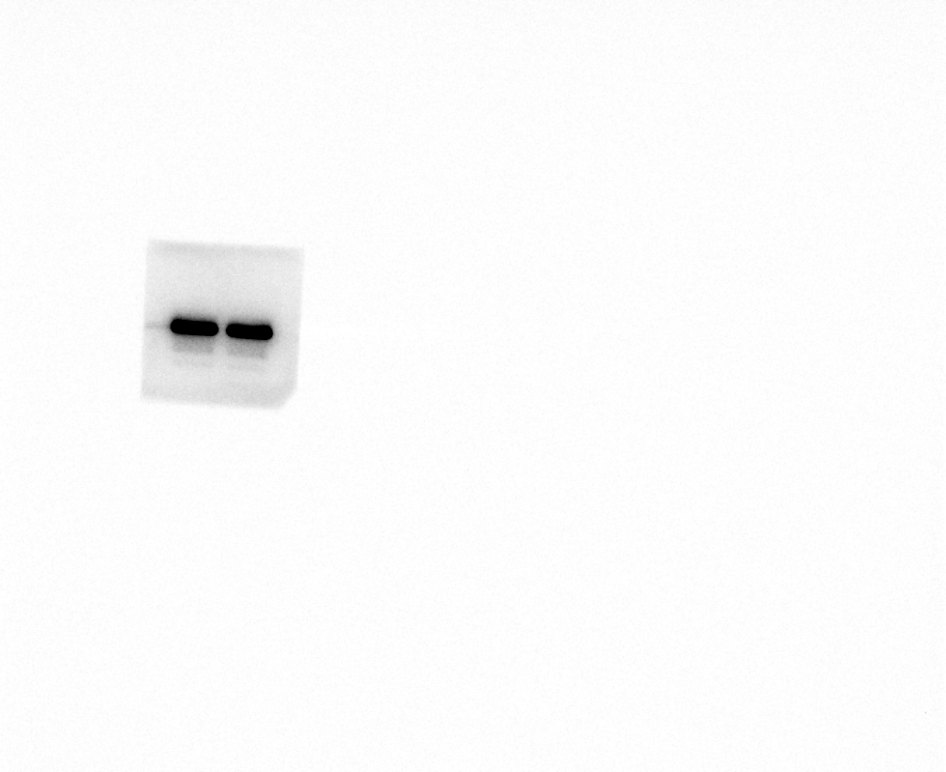

Supplement: Supplementary file 2 [file DataSheet2.ZIP › Original Source Data-Figure 5-7/Figure 5/Figure 5I/GAPDH.tif]

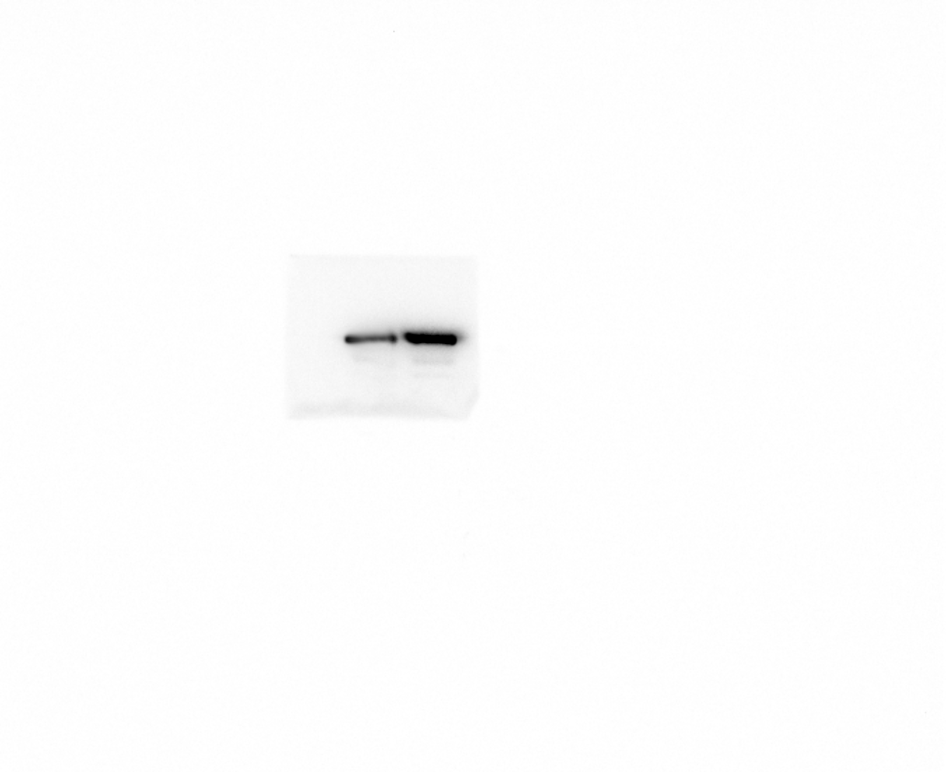

Supplement: Supplementary file 2 [file DataSheet2.ZIP › Original Source Data-Figure 5-7/Figure 5/Figure 5I/LBX2.tif]

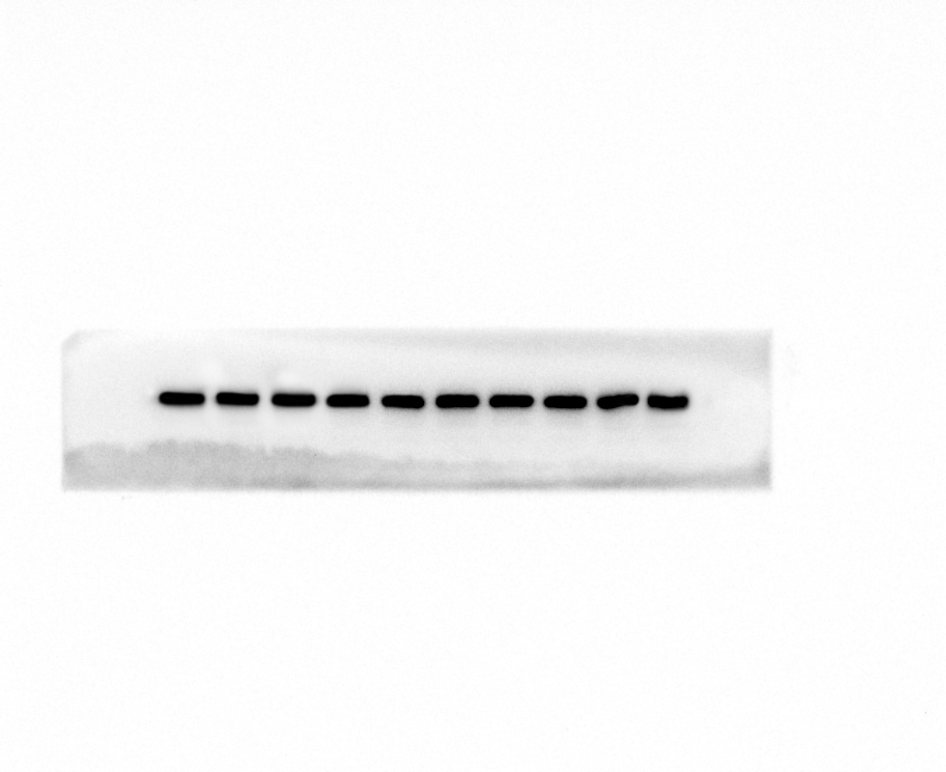

Supplement: Supplementary file 2 [file DataSheet2.ZIP › Original Source Data-Figure 5-7/Figure 6/Figure 6A/GAPDH-NCI-H929.tif]

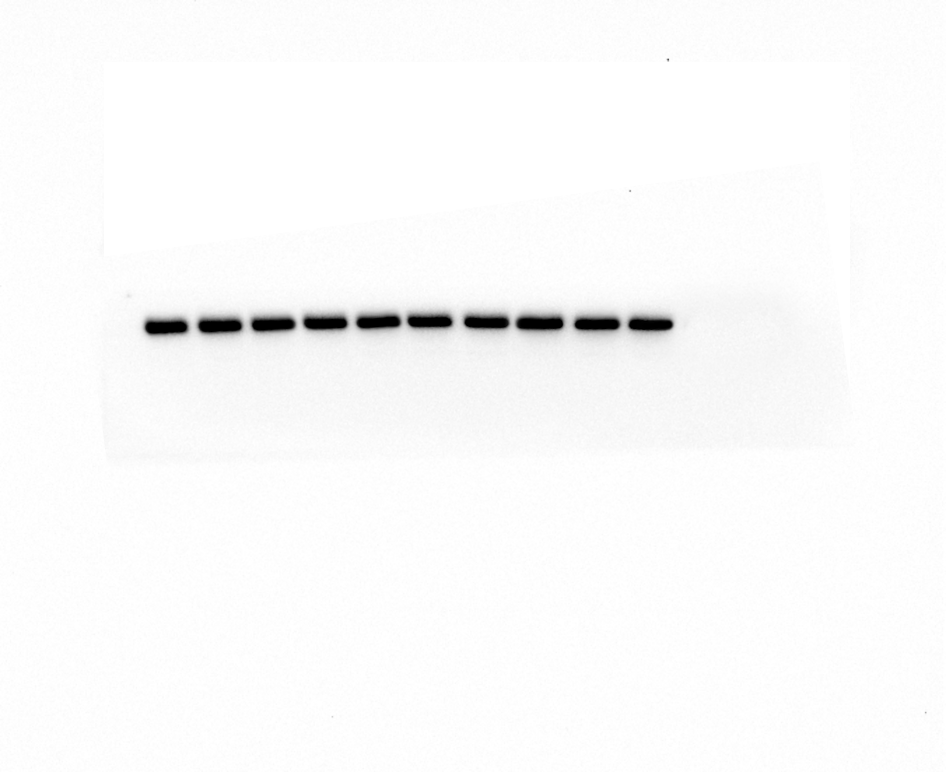

Supplement: Supplementary file 2 [file DataSheet2.ZIP › Original Source Data-Figure 5-7/Figure 6/Figure 6A/GAPDH-U266.tif]

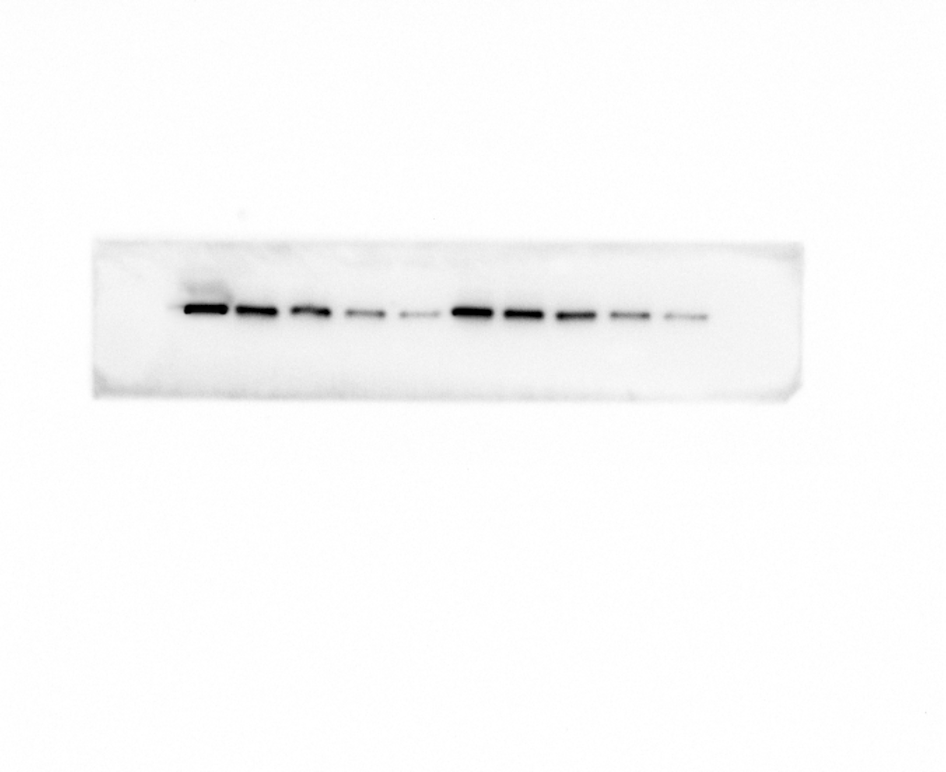

Supplement: Supplementary file 2 [file DataSheet2.ZIP › Original Source Data-Figure 5-7/Figure 6/Figure 6A/LBX2-NCI-H929.tif]

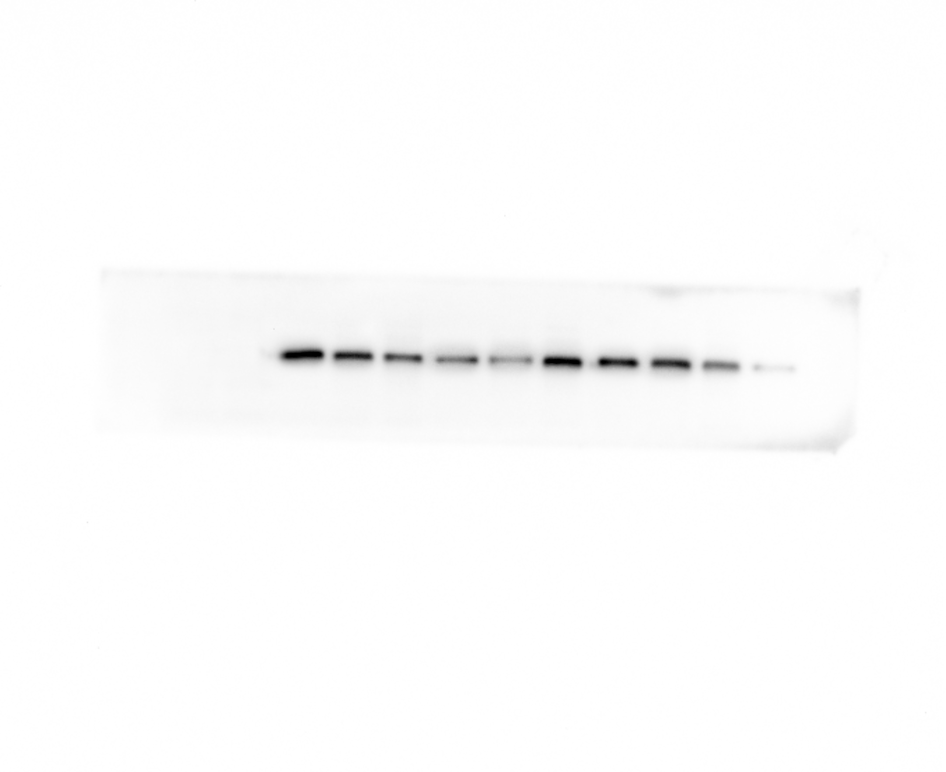

Supplement: Supplementary file 2 [file DataSheet2.ZIP › Original Source Data-Figure 5-7/Figure 6/Figure 6A/LBX2-U266.tif]

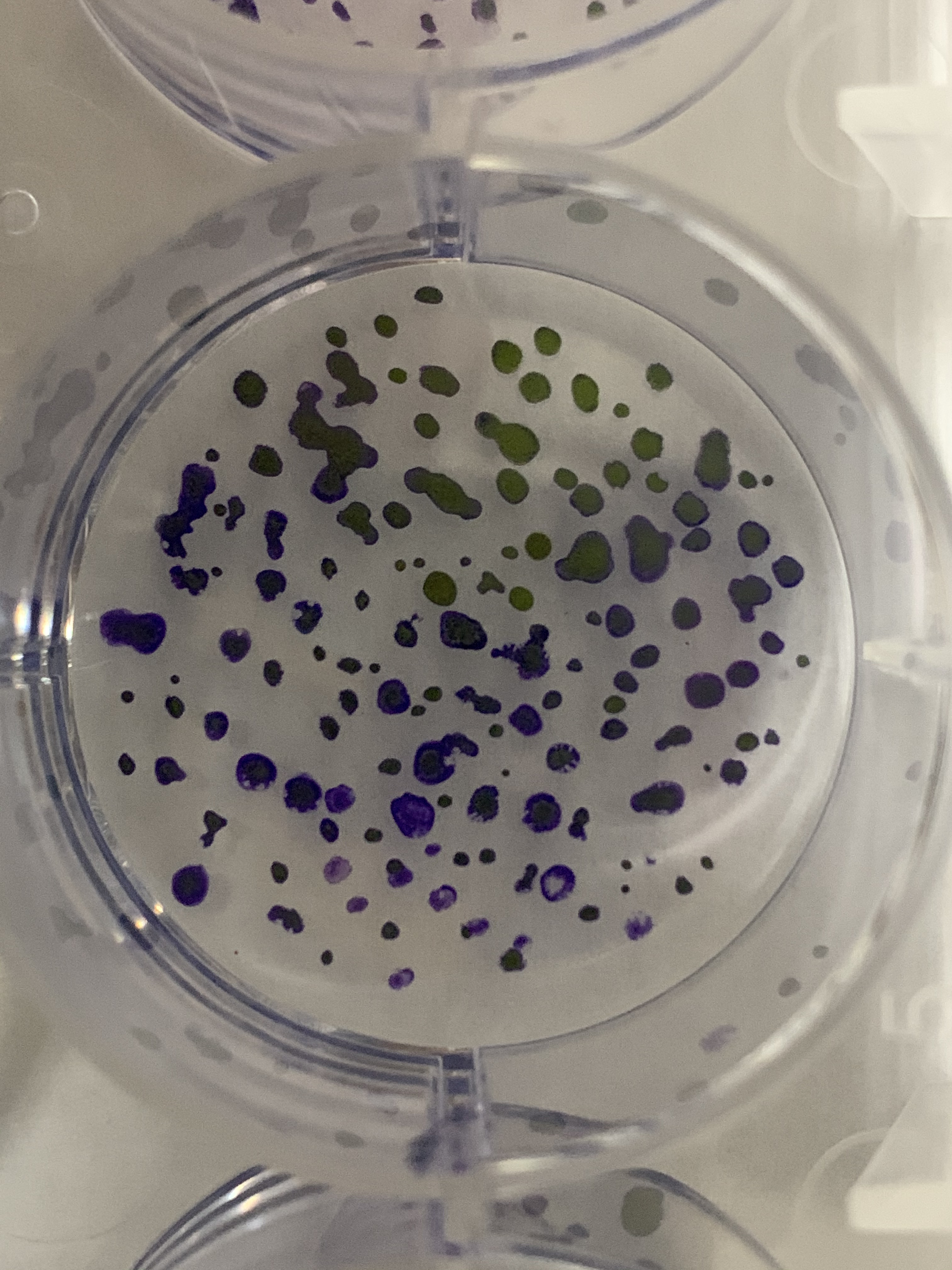

Supplement: Supplementary file 2 [file DataSheet2.ZIP › Original Source Data-Figure 5-7/Figure 7/Figure 7A/H929/Control.JPG]

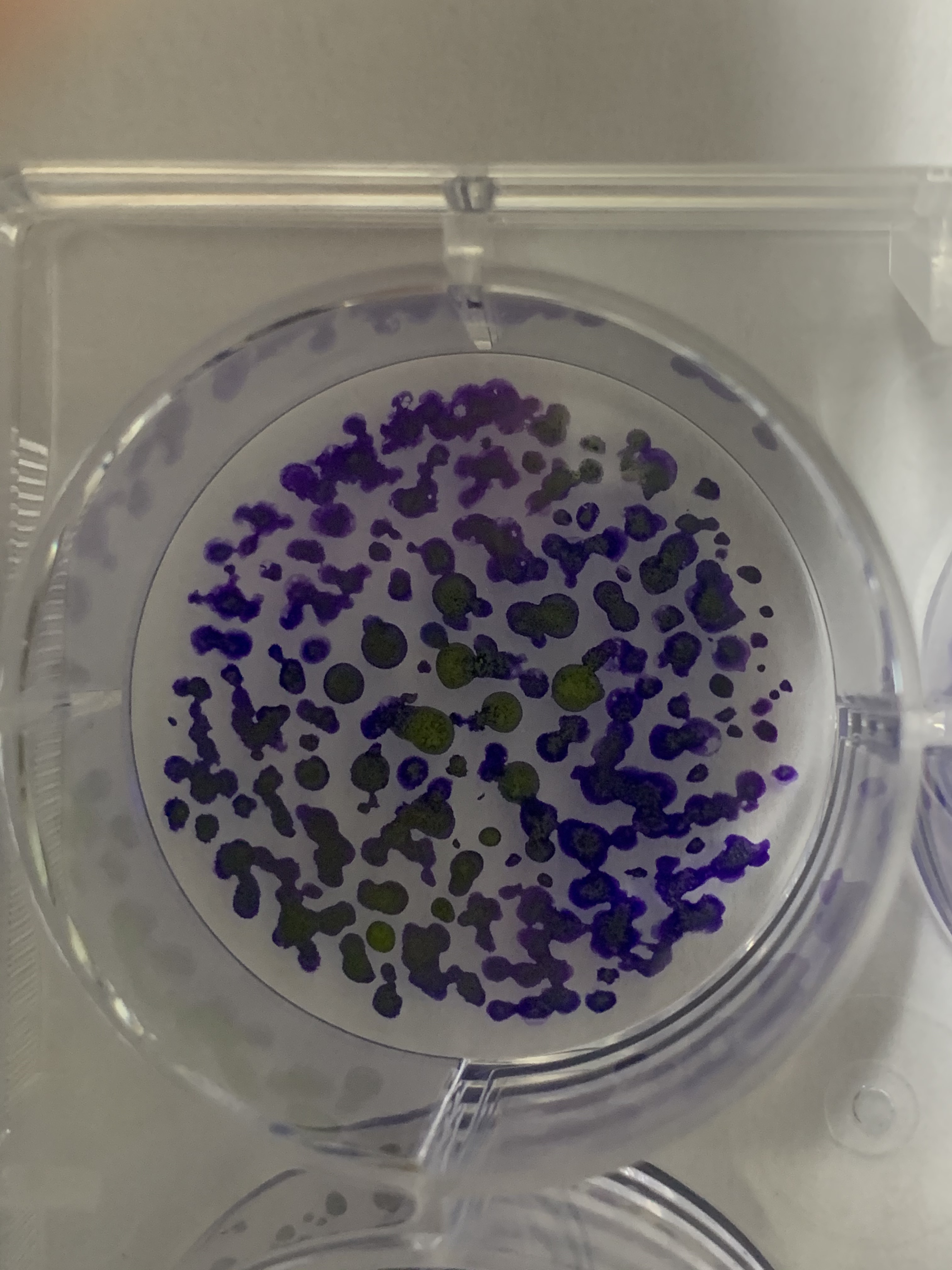

Supplement: Supplementary file 2 [file DataSheet2.ZIP › Original Source Data-Figure 5-7/Figure 7/Figure 7A/H929/LBX2.JPG]

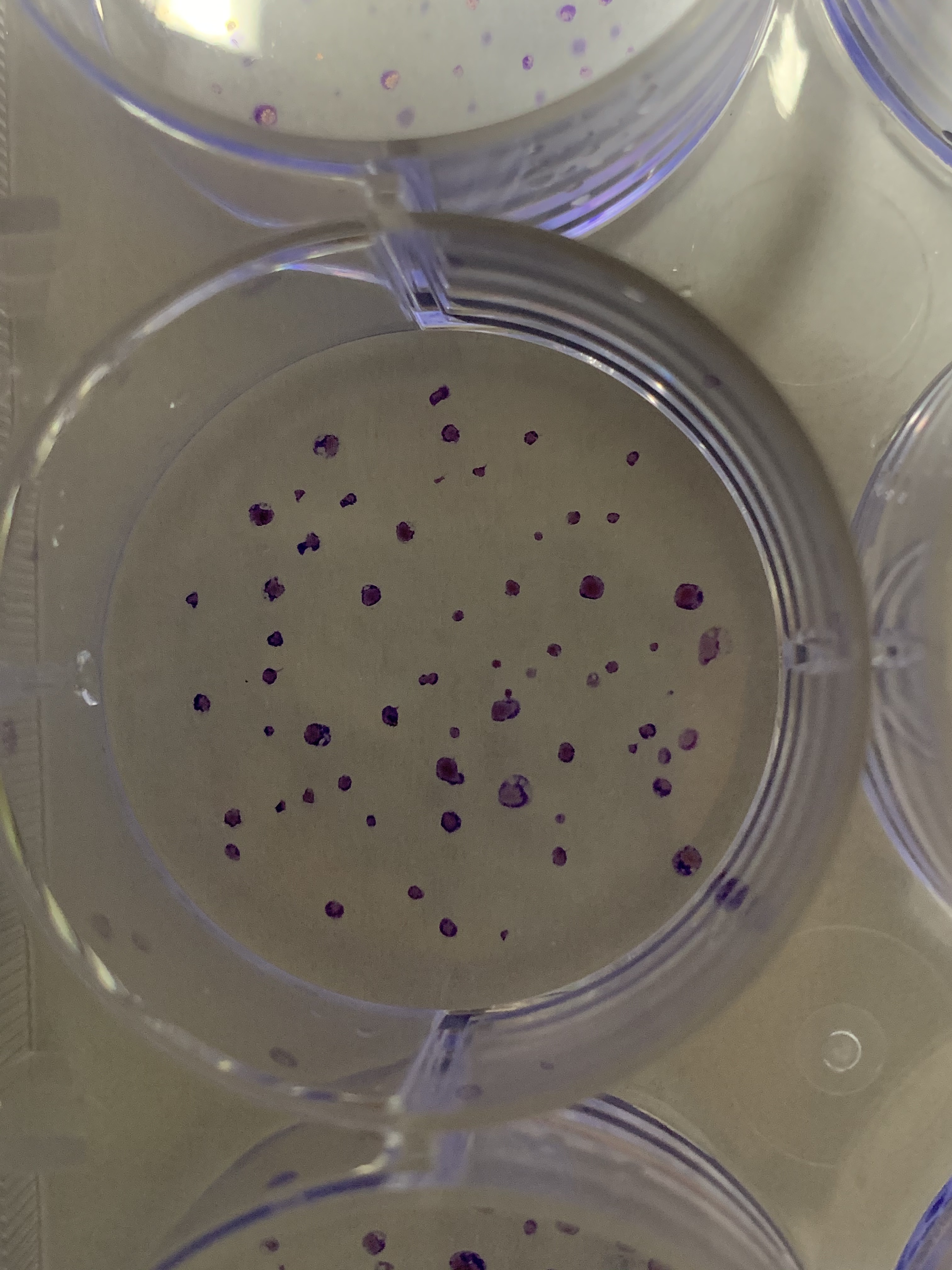

Supplement: Supplementary file 2 [file DataSheet2.ZIP › Original Source Data-Figure 5-7/Figure 7/Figure 7A/H929/shLBX2-AS1+LBX2.JPG]

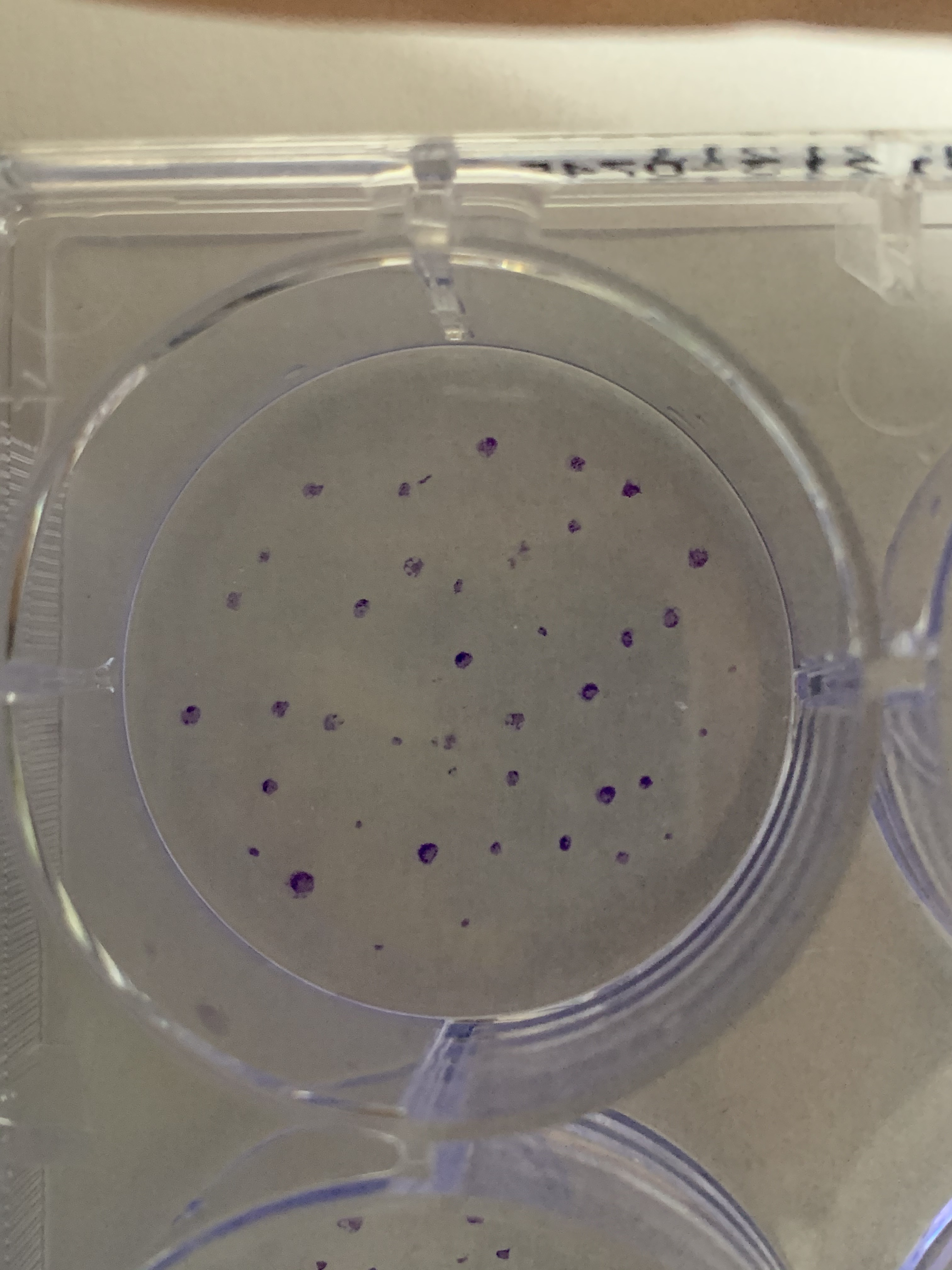

Supplement: Supplementary file 2 [file DataSheet2.ZIP › Original Source Data-Figure 5-7/Figure 7/Figure 7A/H929/shLBX2-AS1.JPG]

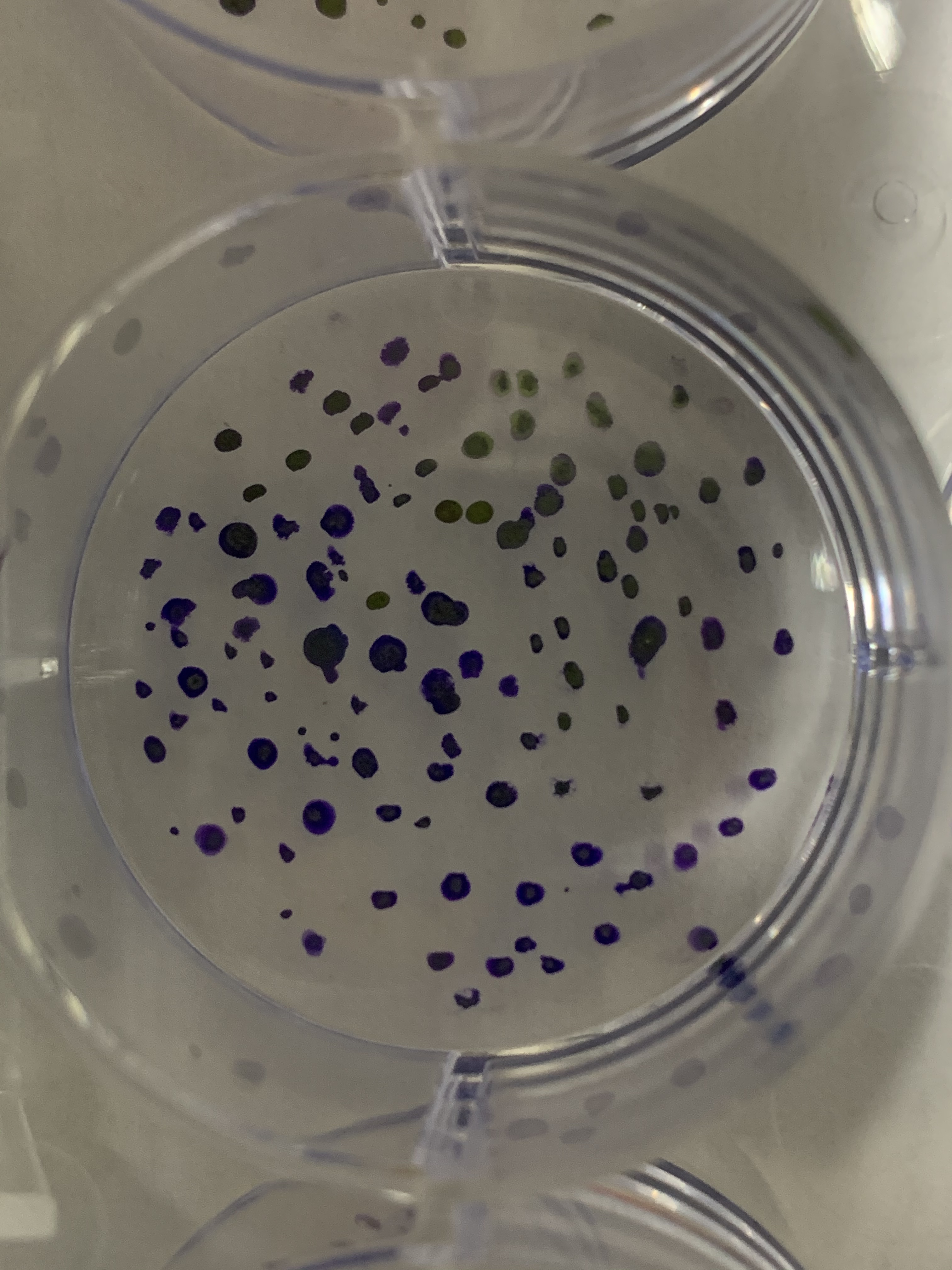

Supplement: Supplementary file 2 [file DataSheet2.ZIP › Original Source Data-Figure 5-7/Figure 7/Figure 7A/U266/Control.JPG]

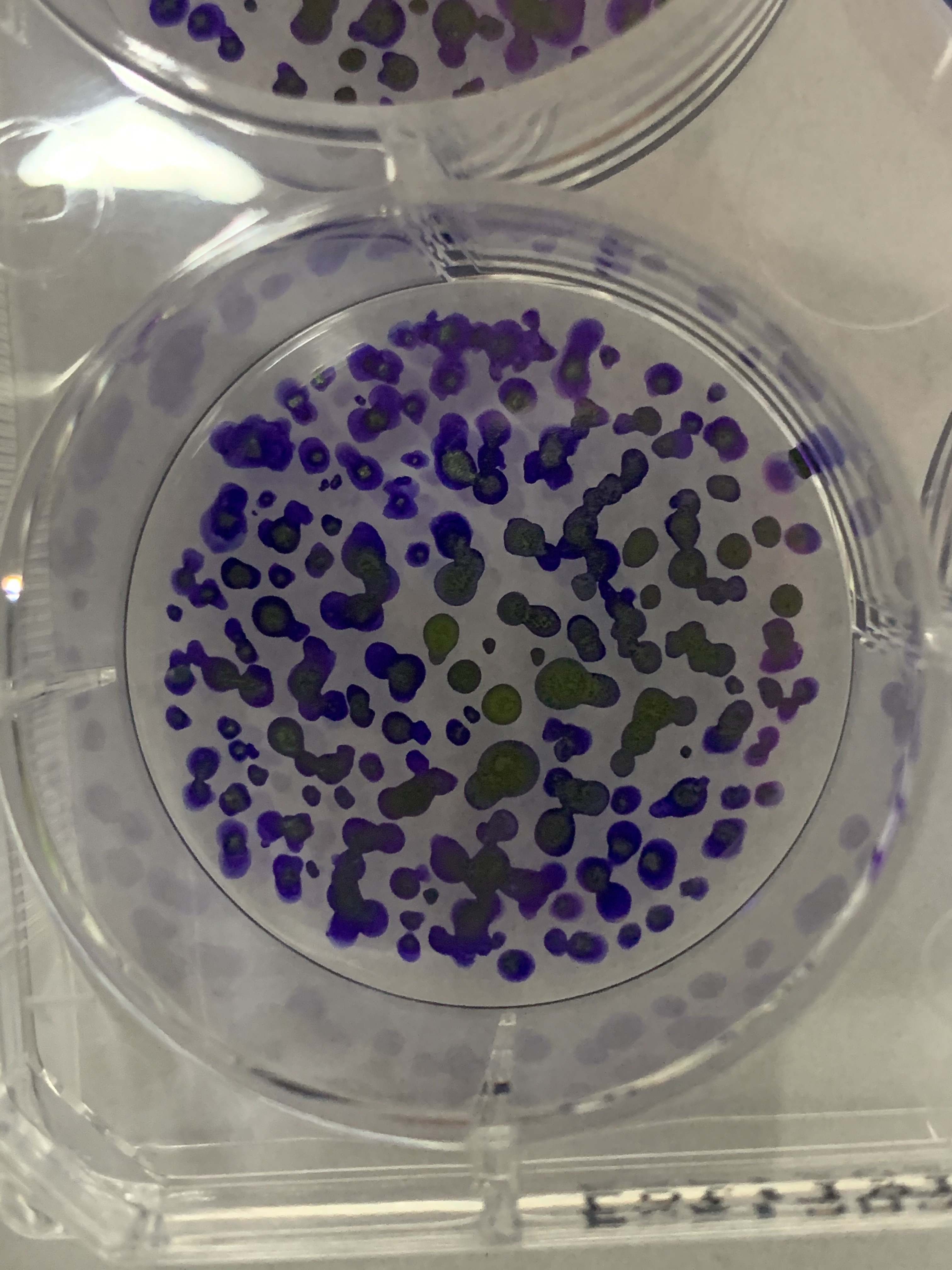

Supplement: Supplementary file 2 [file DataSheet2.ZIP › Original Source Data-Figure 5-7/Figure 7/Figure 7A/U266/LBX2.JPG]

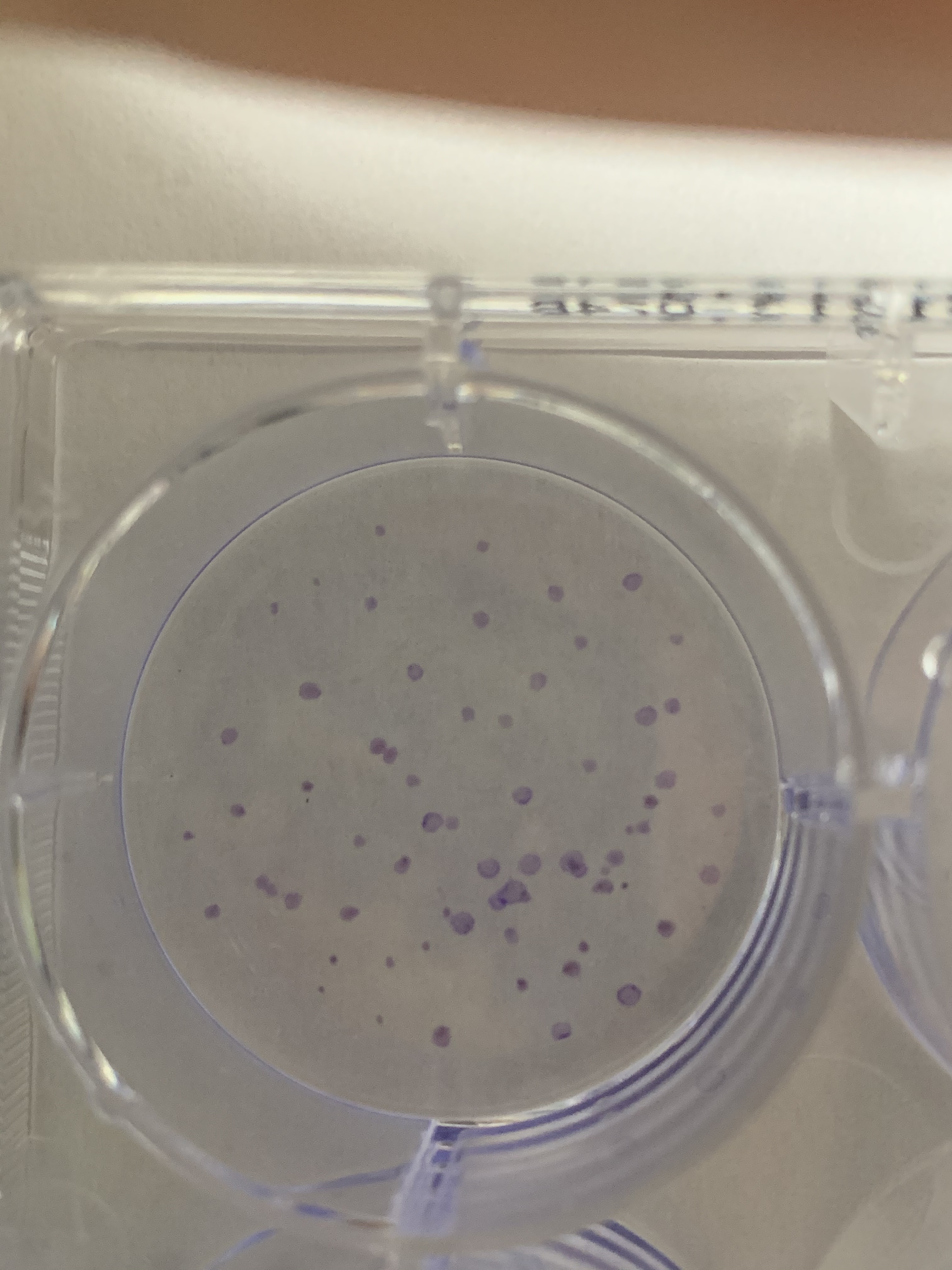

Supplement: Supplementary file 2 [file DataSheet2.ZIP › Original Source Data-Figure 5-7/Figure 7/Figure 7A/U266/shLBX1-AS1+LBX2.JPG]

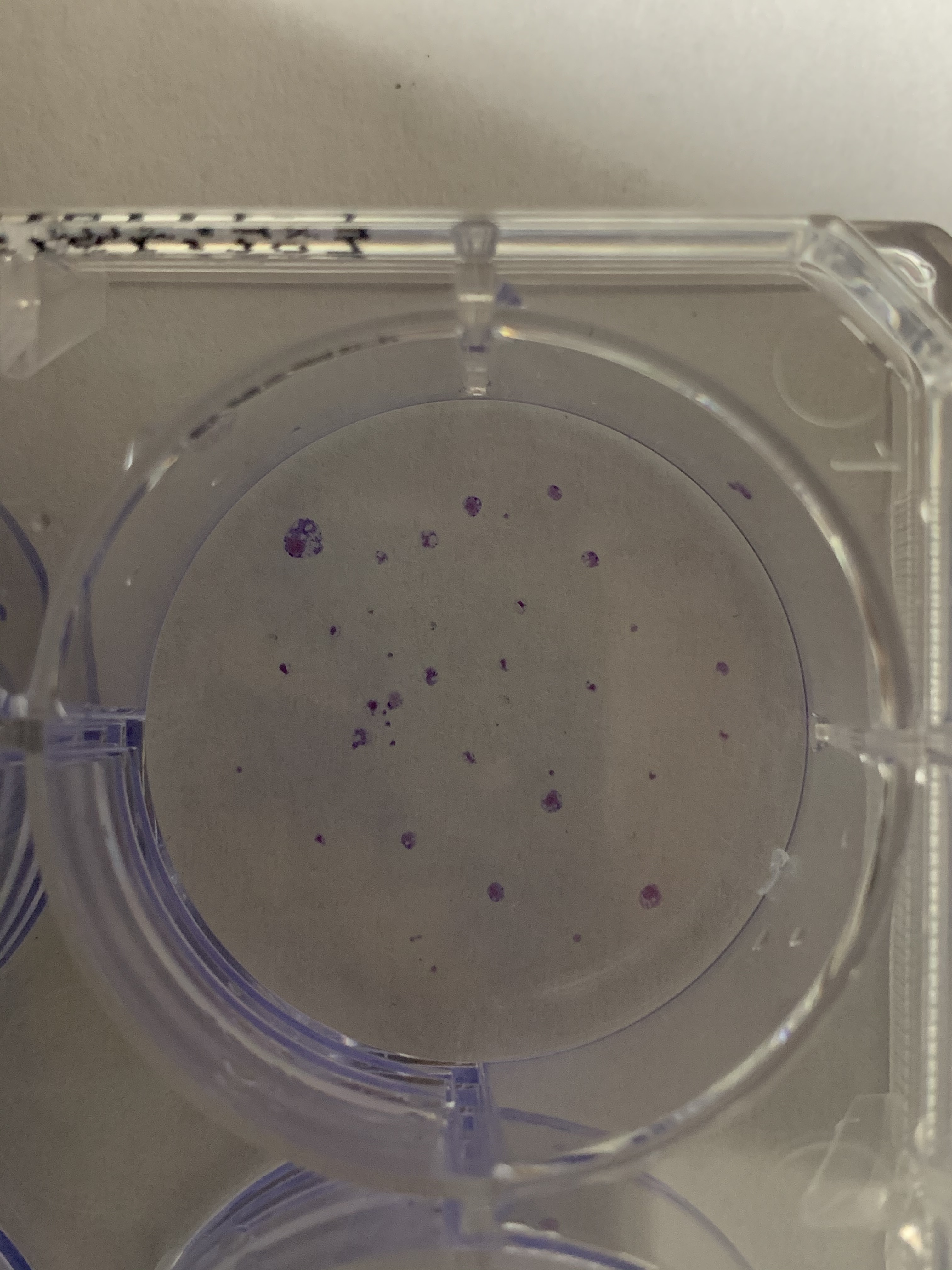

Supplement: Supplementary file 2 [file DataSheet2.ZIP › Original Source Data-Figure 5-7/Figure 7/Figure 7A/U266/shLBX1-AS1.JPG]

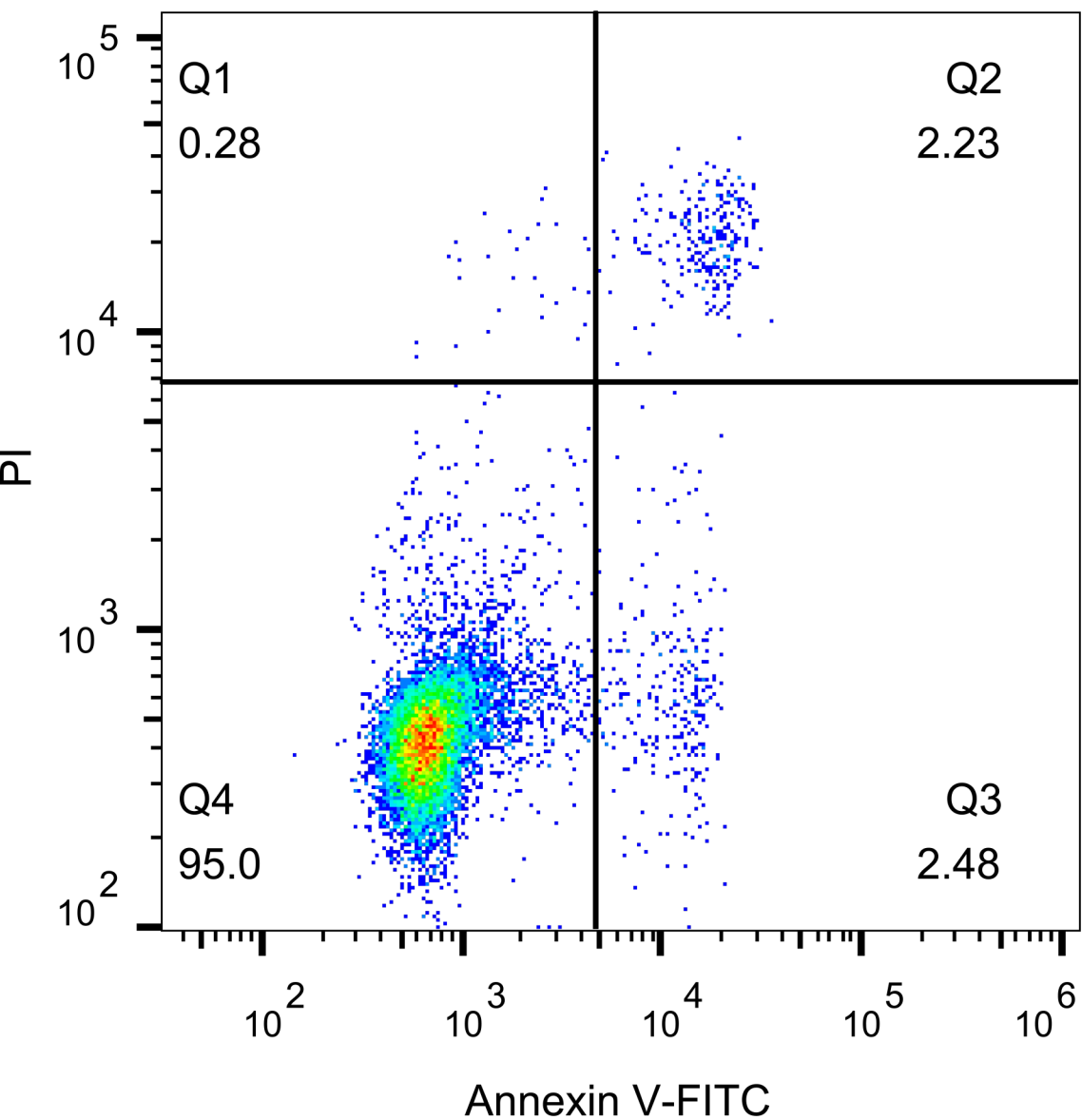

3.fcs

Single Cells

11178

Supplement: Supplementary file 2 [file DataSheet2.ZIP › Original Source Data-Figure 5-7/Figure 7/Figure 7D/NCI-H929-control.pdf]

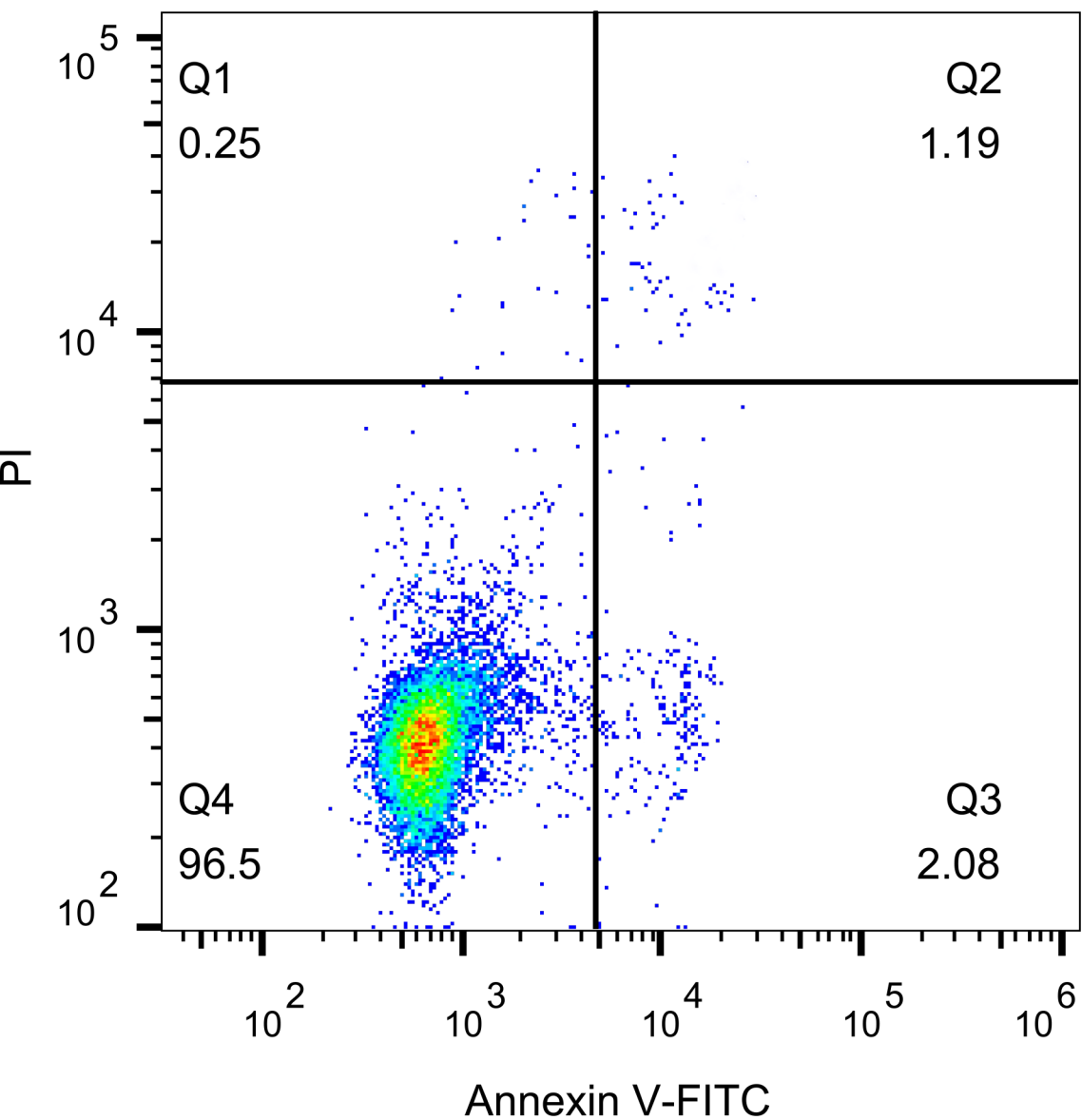

1.fcs

Single Cells

11465

Supplement: Supplementary file 2 [file DataSheet2.ZIP › Original Source Data-Figure 5-7/Figure 7/Figure 7D/NCI-H929-LBX2.pdf]

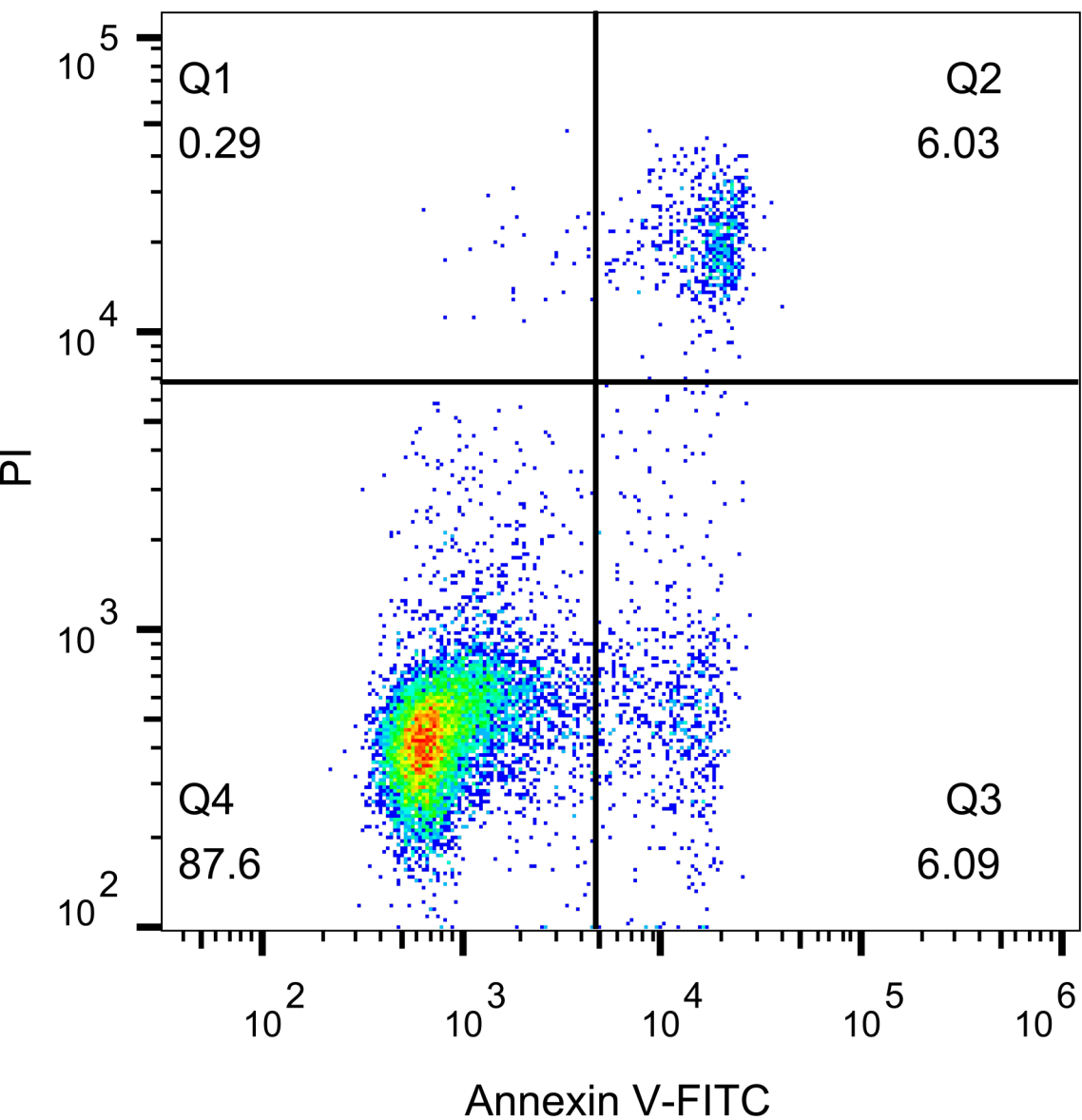

5.fcs

Single Cells

10870

Supplement: Supplementary file 2 [file DataSheet2.ZIP › Original Source Data-Figure 5-7/Figure 7/Figure 7D/NCI-H929-shLBX2-AS1 + LBX2.pdf]

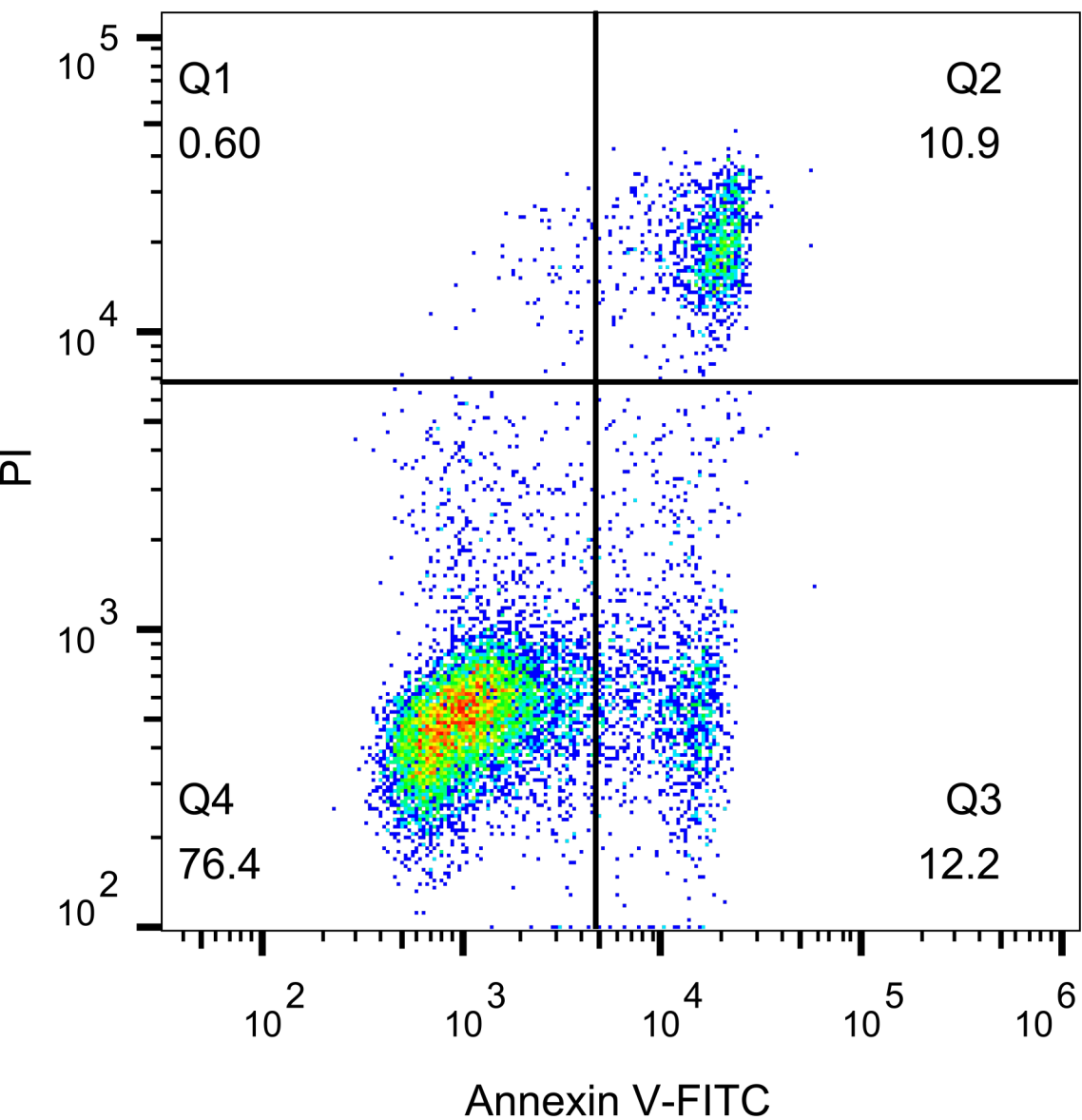

8.fcs

Single Cells

10884

Supplement: Supplementary file 2 [file DataSheet2.ZIP › Original Source Data-Figure 5-7/Figure 7/Figure 7D/NCI-H929-shLBX2-AS1.pdf]

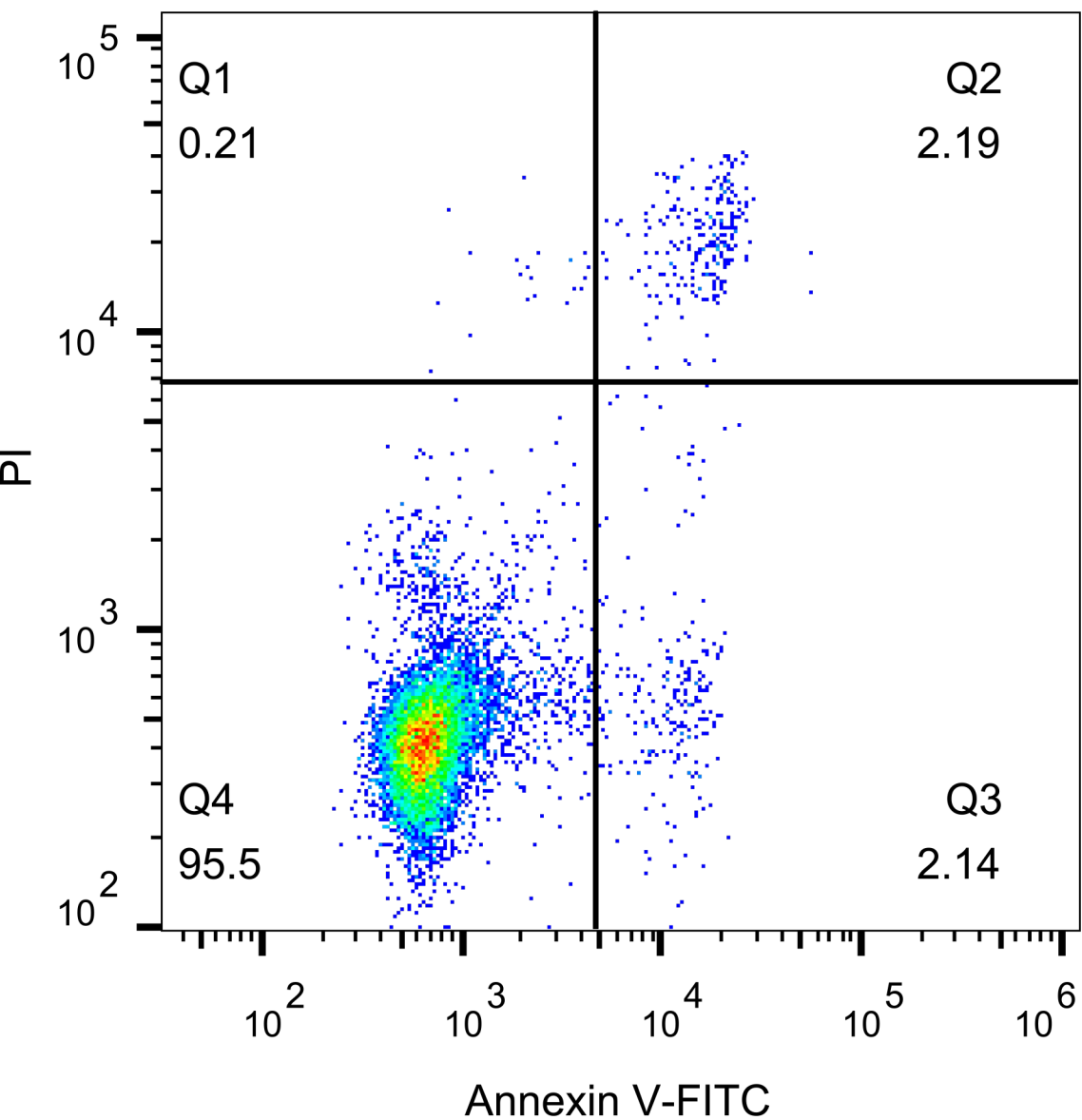

2.fcs

Single Cells

10590

Supplement: Supplementary file 2 [file DataSheet2.ZIP › Original Source Data-Figure 5-7/Figure 7/Figure 7D/U266-control.pdf]

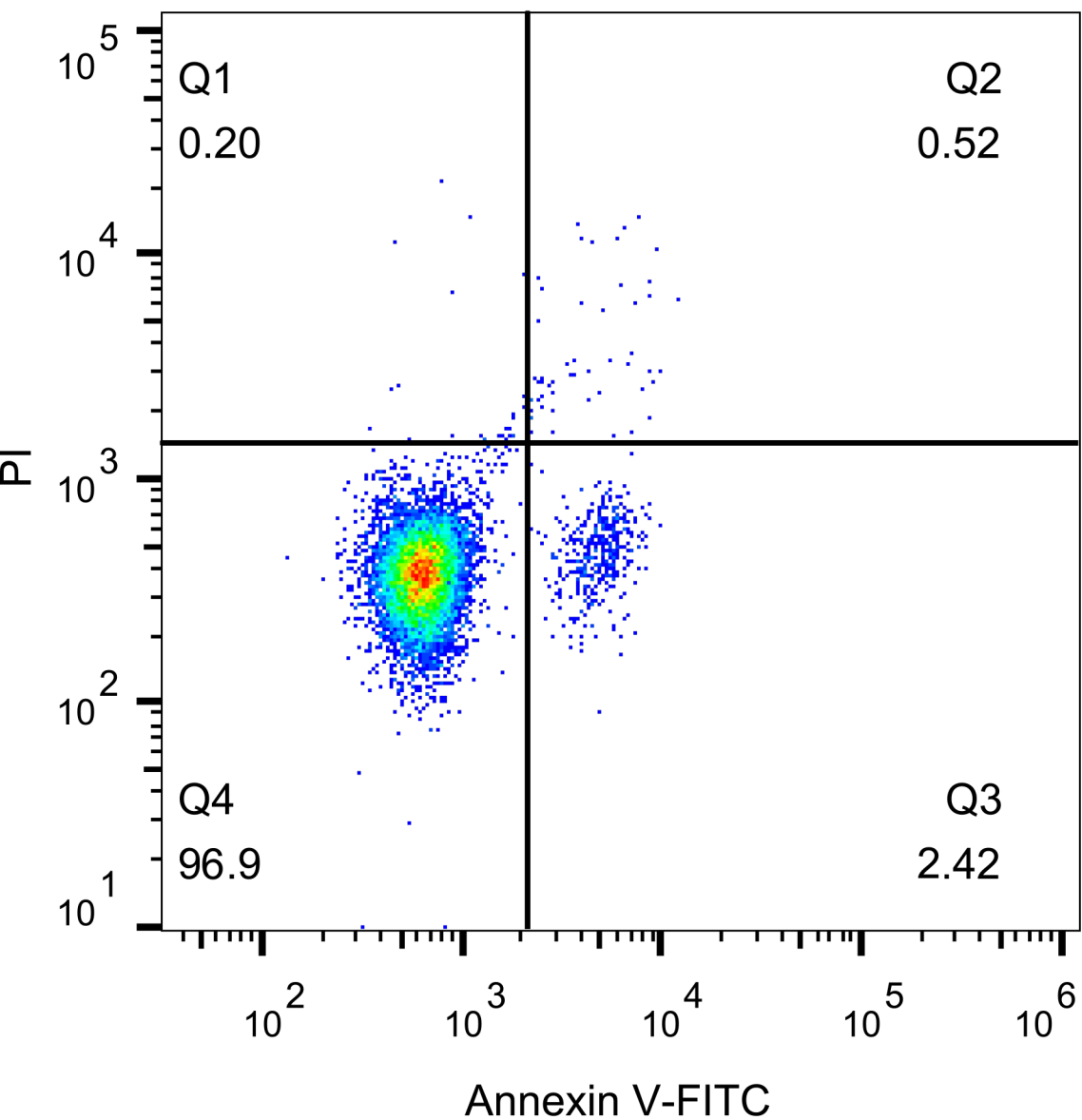

3-2.fcs

Single Cells

11156

Supplement: Supplementary file 2 [file DataSheet2.ZIP › Original Source Data-Figure 5-7/Figure 7/Figure 7D/U266-LBX2.pdf]

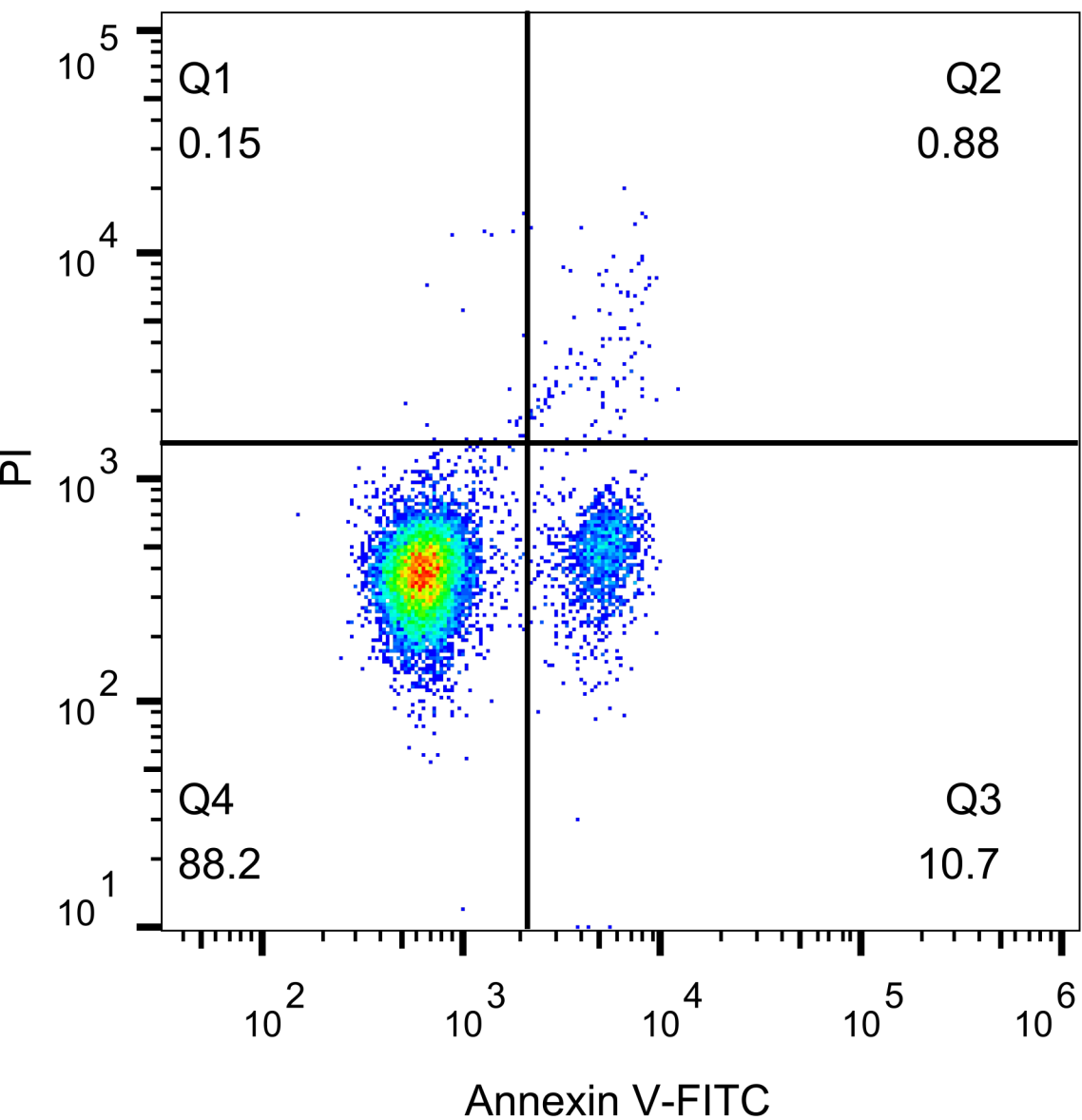

10-1.fcs

Single Cells

12329

Supplement: Supplementary file 2 [file DataSheet2.ZIP › Original Source Data-Figure 5-7/Figure 7/Figure 7D/U266-shLBX2-AS1 + LBX2.pdf]

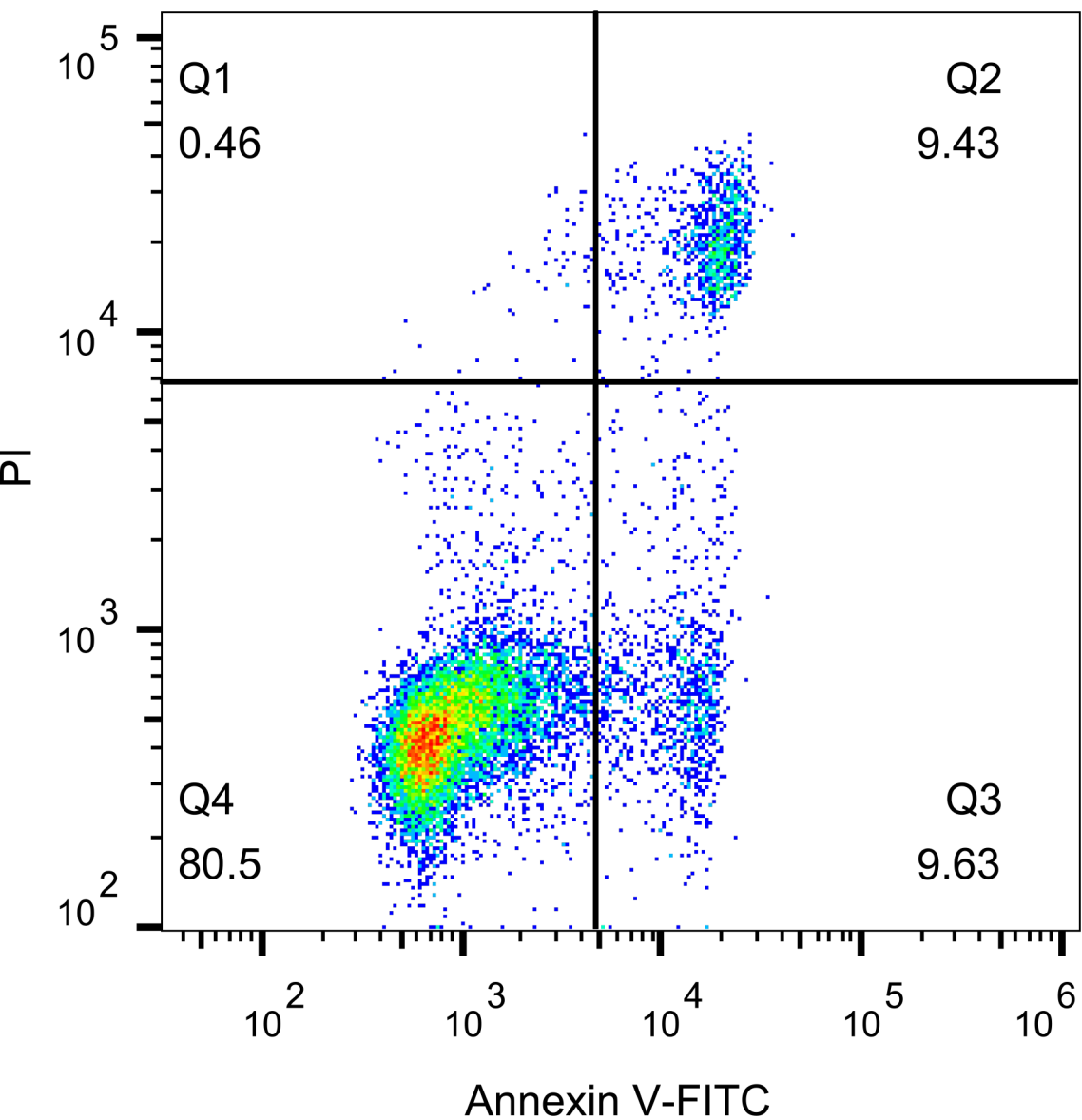

9.fcs

Single Cells

12011

Supplement: Supplementary file 2 [file DataSheet2.ZIP › Original Source Data-Figure 5-7/Figure 7/Figure 7D/U266-shLBX2-AS1.pdf]
